# Metabolic adaptations of *Shewanella eurypsychrophilus* YLB-09 for survival in the high-pressure environment of the deep sea

**DOI:** 10.3389/fmicb.2024.1467153

**Published:** 2024-10-17

**Authors:** Xu Qiu, Xixiang Tang

**Affiliations:** ^1^State Key Laboratory Breeding Base of Marine Genetic Resources, Key Laboratory of Marine Genetic Resources, Fujian Key Laboratory of Marine Genetic Resources, Third Institute of Oceanography, Ministry of Natural Resources, Xiamen, China; ^2^Key Laboratory for Chemical Biology of Fujian Province, MOE Key Laboratory of Spectrochemical Analysis and Instrumentation, College of Chemistry and Chemical Engineering, Xiamen University, Xiamen, China

**Keywords:** deep-sea bacteria, *Shewanella*, high pressure, metabolic regulation, transcriptomic analysis

## Abstract

Elucidation of the adaptation mechanisms and survival strategies of deep-sea microorganisms to extreme environments could provide a theoretical basis for the industrial development of extreme enzymes. There is currently a lack of understanding of the metabolic adaptation mechanisms of deep-sea microorganisms to high-pressure environments. The objective of this study was to investigate the metabolic regulatory mechanisms enabling a strain of the deep-sea bacterium *Shewanella eurypsychrophilus* to thrive under high-pressure conditions. To achieve this, we used nuclear magnetic resonance-based metabolomic and RNA sequencing-based transcriptomic analyses of *S. eurypsychrophilus* strain YLB-09, which was previously isolated by our research group and shown to be capable of tolerating high pressure levels and low temperatures. We found that high-pressure conditions had pronounced impacts on the metabolic pattern of YLB-09, as evidenced by alterations in energy, amino acid, and glycerolipid metabolism, among other processes. YLB-09 adapted to the high-pressure conditions of the deep sea by switching from aerobic intracellular energy metabolism to trimethylamine N-oxide respiration, altering the amino acid profile, and regulating the composition and the fluidity of cell membrane. The findings of our study demonstrate the capacity of microorganisms to alter their metabolism in response to elevated pressure, thereby establishing a foundation for a more profound understanding of the survival mechanisms of life in high-pressure environments.

## Introduction

The most typical environmental factors of the deep sea, which represents 75% of the total ocean area, include low temperatures, darkness, oligotrophicity, and extreme redox gradients in some regions (e.g., hydrothermal and sulfide zones) (Fang et al., 2010; Skropeta and Wei, 2014; Tan et al., 2012). Furthermore, hydrostatic pressure increases with increasing water depth because of the effects of gravity. For every 100 meters of depth, there is a 1 MPa increase in pressure (Saunders and Fofonoff, 1976). Scientific inquiries have established that oceanic areas with hydrostatic pressure exceeding 35 MPa comprise more than 70% of the marine environment (Abe and Horikoshi, 2001). Clearly, the ability to withstand the effects of high hydrostatic pressure represents a considerable challenge for microorganisms attempting to survive in the extreme habitats that characterize the deep sea. It has been postulated that bacteria in extreme environments initiate a reprogramming of gene expression patterns that results in cessation of unnecessary processes, induction of survival-friendly processes, and subsequent resumption of growth at an adaptive rate that aligns with the extreme environmental conditions (Kloska et al., 2020). This indicates that deep-sea microorganisms have evolved metabolic pathways and molecular bases that enable them to cope with the high-pressure environments of the deep sea (Simonato et al., 2006). Studies aimed at elucidating the mechanisms of high-pressure adaptation of deep-sea microorganisms could have considerable impacts on our understanding of microbial adaptation and survival in extreme environments.

Our research group previously isolated a Gram-negative bacterial strain of *Shewanella eurypsychrophilus* (YLB-09) from a deep-sea sediment sample (DY125-40I-SWIR-S038-TVG20; 49.73°W, 37.78°S) collected from the hydrothermal zone of the Southwest Indian Ocean at a water depth of 2,315 m. YLB-09 exhibits pressure-tolerant characteristics, with an optimal pressure of 0.1 MPa and the capability to grow under high pressure (50 MPa). Additionally, the strain displays psychrophilic characteristics, with an optimal temperature of 15°C and a tolerance for low temperatures (4°C). These traits align with those of a typical deep-sea, pressure-tolerant, and psychrophilic bacterial strain.

In a previous study, we analyzed the gene evolution of 41 marine-sourced strains of the genus *Shewanella*. Our findings revealed that the genomic evolution of *Shewanella* strains from the surface, middle, deep, and abyssal zone of the ocean tended to follow a pattern of first gaining genes and then streamlining them. From the shallow to the deep oceanic zones, and subsequently to the abyssal zone, the genomic size of each strain exhibited a dual pattern of gradual expansion and subsequent drastic reduction. The genome of YLB-09 was found to contain numerous genes that are conducive to high-pressure adaptation. These include genes related to flagella and hyphae, cellular respiration, proteins and enzymes, and molecular chaperones (Cao et al., 2022), which are closely associated with the high-pressure adaptation of microorganisms (Xiong et al., 2016; Eloe et al., 2008). Therefore, strain YLB-09 is ideal for studying the mechanisms of high-pressure adaptation of deep-sea microorganisms.

Despite numerous reports on gene and transcriptional regulatory mechanisms for microbial high-pressure adaptation, the metabolic adaptation mechanisms remain poorly understood. In this study, we conducted experiments on strain YLB-09 in simulated deep-sea high-pressure and low-temperature environments. We also employed nuclear magnetic resonance (NMR)-based metabolomics and RNA sequencing (RNAseq)-based transcriptomics analyses to investigate the metabolic regulatory mechanisms of strain YLB-09 under high pressure. The findings of our study provide a theoretical basis for further understanding of high-pressure adaptation mechanisms in deep-sea microorganisms.

## Materials and methods

### Experimental design and microbial culture protocols

The bacterial strain *S. eurypsychrophilus* YLB-09 (KCTC 62910 = MCCC 1A12717) was initially isolated and preserved by our research group. The growth curves of strain YLB-09 at different temperatures and pressures were obtained previously, and the optimal growth temperature and pressure were determined to be 15°C and 0.1 MPa, respectively (Supplementary Figure S1).

The experimental design stipulated that YLB-09 cells were cultured under the following conditions. For normal pressure culture, an overnight culture of YLB-09 grown under 15°C and 0.1 MPa was inoculated into 50 mL of sterile 2216E liquid medium (Haibo, China) at a ratio of 1:100 and transferred to an empty, sterile, NaCl injection bag. A fluoride solution (Fluorinert FC-40, Belgium) was oxygenated to saturation (>2 h) using an oxygen cylinder, and was sterilized by filtration. A sterile syringe was used to inject the oxygenated fluoride solution into the injection bag containing the bacterial solution at a ratio of 4:1 by volume. The NaCl injection bag was then placed in two aliquots, and cultured in a light-protected incubator at 4°C (NPLT group, *n* = 8) or at 15°C (NPOT group, *n* = 8). For high pressure culture, the sample preparation was conducted in a manner consistent with normal pressure culture, with the exception of placing the NaCl injection bag in a high-pressure vessel (Nantong Feiyu, China) with the pressure set at 23 MPa to simulate a marine environment characterized by elevated hydrostatic pressure. The high-pressure culture vessel was placed in an incubator at 4°C (HPLT, *n* = 10) and 15°C (HPOT, *n* = 10) until sample collection.

### Bacterial collection and quenching

When cultures reached the mid-logarithmic growth phase (optical density at 600 nm: 0.6 for NPOT, 0.5 for HPOT, 0.7 for NPLT, 0.5 for HPLT), the bacteria were collected by centrifugation at 4°C and 9,800 × g for 5 min. Next, 10 mL of a solution comprising 60% methanol (−80°C) and 0.85% (w/v) NaCl, in a 3:2 volume ratio, was added to each sample on ice for resuspension and the cell samples were placed in a −80°C freezer for 30 min to stop metabolic processes, then collected by centrifugation at 4°C, 9,800 × g for 5 min. The supernatant was removed and the cells were resuspended in 10 mL phosphate-buffered saline (PBS, 0.01 M) and collected by centrifugation (4°C, 9,800 × g, 5 min) three times in preparation for the subsequent metabolite extraction.

### Extraction of water-soluble metabolites

Following quenching on ice, the bacteria were subjected to ultrasonic disruption using an ultrasonic cell crusher (SM-900D, Sunmax Technology, China). A 50-mL methanol:water mixture (10:9 volume ratio) that was previously cooled on ice was added to the aforementioned cell precipitates, and sonication was conducted at 300 W for 2 s with a 3-s pause, for a total of 15 min. Following sonication, 5 mL of ice-precooled chloroform was added to the mixture, which was then inverted to ensure thorough homogenization. The mixture was then subjected to another 10 min of sonication at 300 W (2-s sonication, 3-s pause). The sonicated mixture was subjected to centrifugation for 10 min at 4°C and 9,800 × g, producing a layered appearance. The upper layer was extracted for subsequent analysis.

### NMR sample preparation

The methanol in the aforementioned solution was desiccated by slowly passing nitrogen into the collected aqueous solution of metabolites. The methanol-removed solution was transferred to a freeze dryer for lyophilization to remove the solvent. NMR buffer (50 mM PBS, 0.025 mM trimethylsilylpropanoic acid) was added to the lyophilized metabolite samples, which were then vortexed and shaken to ensure homogeneous mixing, and collected by centrifugation (4°C, 9,800 × g, 5 min). Each sample was transferred to a 5-mm NMR tube for subsequent analysis.

### NMR spectra acquisition

The ^1^H-NMR spectra were acquired using a Bruker Avance III 600 MHz instrument with a noesygppr1d (relaxation delay-90°-t1-90°-τm-90°-acq) pulse sequence and an ambient temperature of 25°C. The data acquisition time (Tacq) was 2.66 s, the relaxation delay time (d1) was 4 s, the number of acquisition points (TD) was 64 K, the spectral width (SW) was 15.000 ppm, the number of null pickup scans (DS) was 4, and the number of accumulation scans (NS) was 64. Data acquisition was accompanied by suppression of the water peaks.

To improve the accuracy and reliability of metabolite fingerprinting, the 2D ^1^H-^1^H total correlation spectroscopy (TOCSY) spectra were acquired using a Bruker Avance III 600 MHz instrument with a mlevgpph19 pulse sequence at an ambient temperature of 25°C.

### NMR spectra processing and metabolite fingerprinting

The acquired ^1^H-NMR spectra were processed using MestReNova 14 software. Prior to the application of the Fourier transform, window function processing was conducted on the free induction decay (FID) signal using an exponential function, setting the parameter to LB = 0.3 Hz. Subsequently, the 1D proton NMR (^1^H-NMR) spectra underwent manual baseline adjustment and phase correction, as well as calibration with the internal standard tetraphosphate (TSP), with a chemical shift of 0.000 ppm. The spectra were processed in accordance with the aforementioned methodology, concurrently eliminating the peakless spectra beyond 9.000 ppm and the water peaks within the range of chemical shifts *δ*4.700–5.200 ppm. This was done to negate the impact of the water de-repression effect on the spectral line integration.

The 2D ^1^H-^1^H TOCSY spectra were processed using the Bruker companion software TopSpin (version 4.0.8), followed by similar data corrections for baseline and phase, and calibration with lactic acid.

To identify the metabolites in the NMR spectra, we employed the Chenomx NMR Suite software (version 8.3) and the HMDB database.^1^

### Data analysis

The NMR spectra within the range of 0.000–9.000 ppm were integrated in segments, with the range of each integration (binning) set to 0.001 ppm. In addition to NMR spectra fingerprinting, segmented integration data were normalized based on the trimethylsilylpropanoic acid (TSP) spectrum integration values and the number of viable bacteria using Matlab software (version 2020a). This was done to compensate for sample concentration differences. Normalization of the data providing a means of representing the absolute concentration of each metabolite for subsequent analysis.

The normalized metabolite concentration data were imported into Simca software (version 4.0.8) and subjected to multivariate statistical analyses, including unsupervised principal component analysis (PCA) and supervised partial least squares-discriminant analysis (PLS-DA), following the centering process of Pareto scaling.

Following importation of the metabolite concentration data into SPSS software (version 19), multiple comparisons using Tukey’s test were performed to ascertain significance levels associated with changes in metabolite concentration.

The objective of the screening process was to identify differential metabolites as well as characteristic metabolites. The differential metabolites were obtained from the aforementioned SPSS processing, using a cutoff for the change in concentration of *p* < 0.05. The identification of characteristic metabolites was based on the variable importance in projection (VIP) value of the metabolite and the *p*-value of the between-group difference in concentration. Metabolites were considered characteristic when they exhibited a VIP value >1 and a *p*-value <0.05.

### Metabolic pathway enrichment analysis

Metabolic pathway enrichment analysis was conducted using the MetaboAnalyst Web server (version 5.0, https://www.metaboanalyst.ca/), employing cutoffs of *p* < 0.05 (−log10(*p*) > 1.301) and pathway impact value (PIV) >0.1. This was undertaken to identify metabolic pathways with notable regulation in *S. eurypsychrophilus* in response to different treatments.

### RNA extraction and sample quality testing

Bacterial samples were removed from the −80°C freezer and subjected to quality testing. Three bacterial replicates cultured under each condition were obtained for extraction of RNA on ice using the RNA kit (Takara, China), in accordance with the manufacturer’s instructions.

### Reference genome alignment and gene annotation

*S. eurypsychrophilus* YLB-09 (NCBI accession number: CP045427) was utilized as the reference genome. Sequencing data were aligned to the reference genome using Bowtie software (version 2.29) and following the Burrows–Wheeler method, to obtain reads for subsequent analysis. The protein sequences were uploaded to five major databases [NCBI non-redundant (NR), Swiss-Prot, Pfam, Clusters of Orthologous Genes (COG), and Gene Ontology (GO)] for comparison, and the transcriptome gene sequences and related metabolic pathways were subjected to functional annotation.

Quantitative gene expression analysis was performed using RSEM software,^2^ which was employed to calculate the expression levels of genes and transcripts for each sample. Gene length and sequencing depth were standardized to ensure consistent total sample expression, using transcripts per million reads (TPM) as a quantitative metric.

Venn analysis was performed to determine the numbers of shared and unique genes. Correlation analysis was employed to ascertain both the consistency of biological replicates within each group of samples and the variability of gene expression between samples. Genes exhibiting differential expression were subsequently annotated and analyzed using the Kyoto Encyclopedia of Genes and Genomes (KEGG) database. The top 20 items in the KEGG enrichment analysis were compared, among which the metabolism-related items were screened for correlation with the metabolomics results.

### Metabolomic and transcriptomic data interaction analysis

Pathways associated with the differentially expressed genes (DEGs) were identified using the KEGG pathway mapping website^3^ for annotation, employing information on upregulation or downregulation of metabolite concentrations and gene expression levels, and then mapped to the metabolomics-enriched pathways. Each candidate regulated pathway was analyzed for corresponding changes in the expression of both metabolites and genes within the pathway.

## Results

### ^1^H-NMR spectra of strain YLB-09 under different conditions

**Figure 1:**
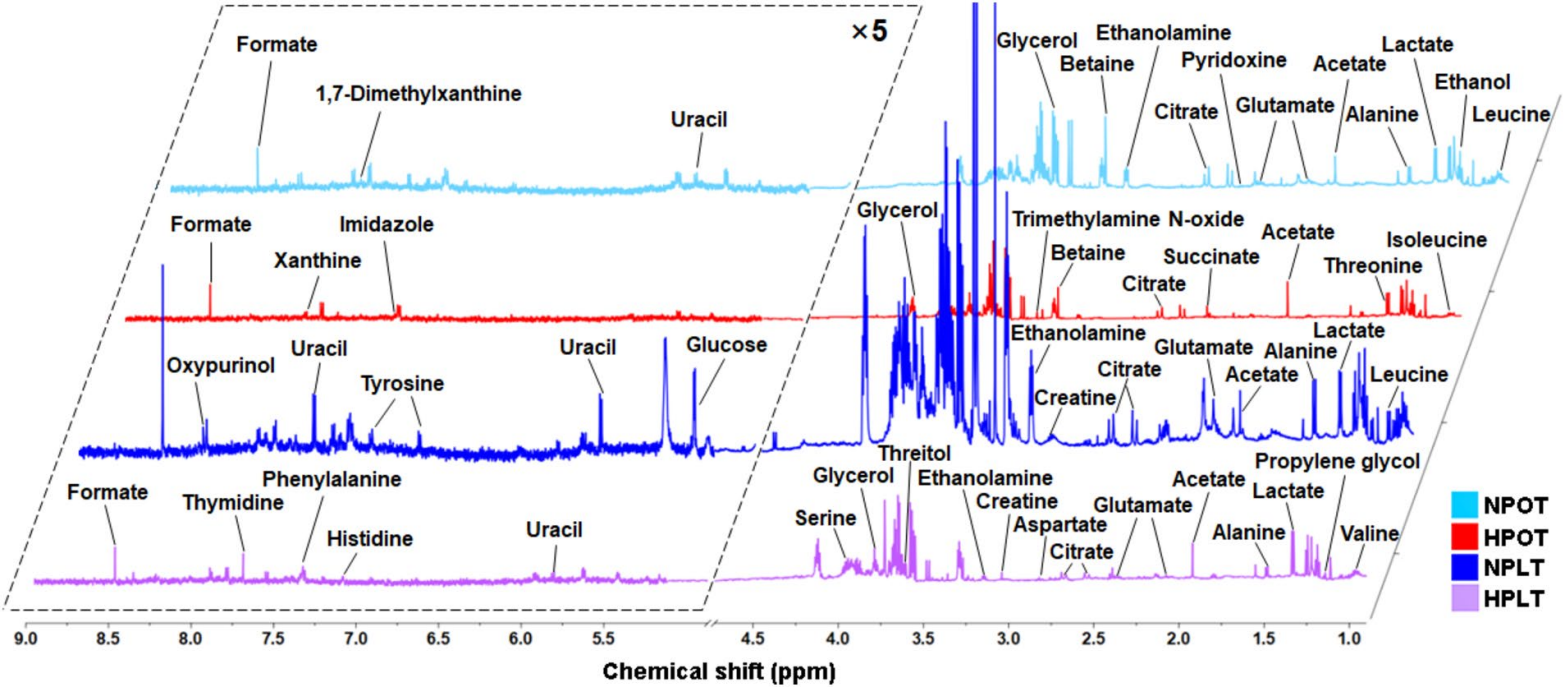
Averaged 1D ^1^H-NMR spectra recorded on aqueous extracts derived from the NPOT, HPOT, NPLT and HPLT groups of YLB-09 cells. The NMR spectra were recorded on a 600 MHz NMR spectrometer at 25°C. Vertical scales were kept constant in the four spectra. The resonance region of 0.900–9.000 ppm is shown, whereas the resonance region of 4.700–5.200 ppm (water) was removed. For the purpose of clarity, the resonance region of 5.200–9.000 ppm has been magnified five times compared to the other region of 0.900–4.700 ppm.

A total of 34 intracellular water-soluble metabolites of YLB-09 were identified in the ^1^H-NMR spectra (Figure 1 and Supplementary Table S1). Subsequently, the results of the 1D ^1^H-NMR spectra of the identified metabolites were confirmed using the 2D ^1^H-^1^H TOCSY spectra (Supplementary Figure S2). These 34 metabolites were classified into four main groups: (1) metabolites related to amino acid metabolism; (2) metabolites related to the metabolism of carbohydrates and their derivatives; (3) metabolites related to the metabolism of nucleotides; and (4) metabolites related to other metabolic processes.

### Multivariate statistical analysis of metabolic patterns

**Figure 2:**
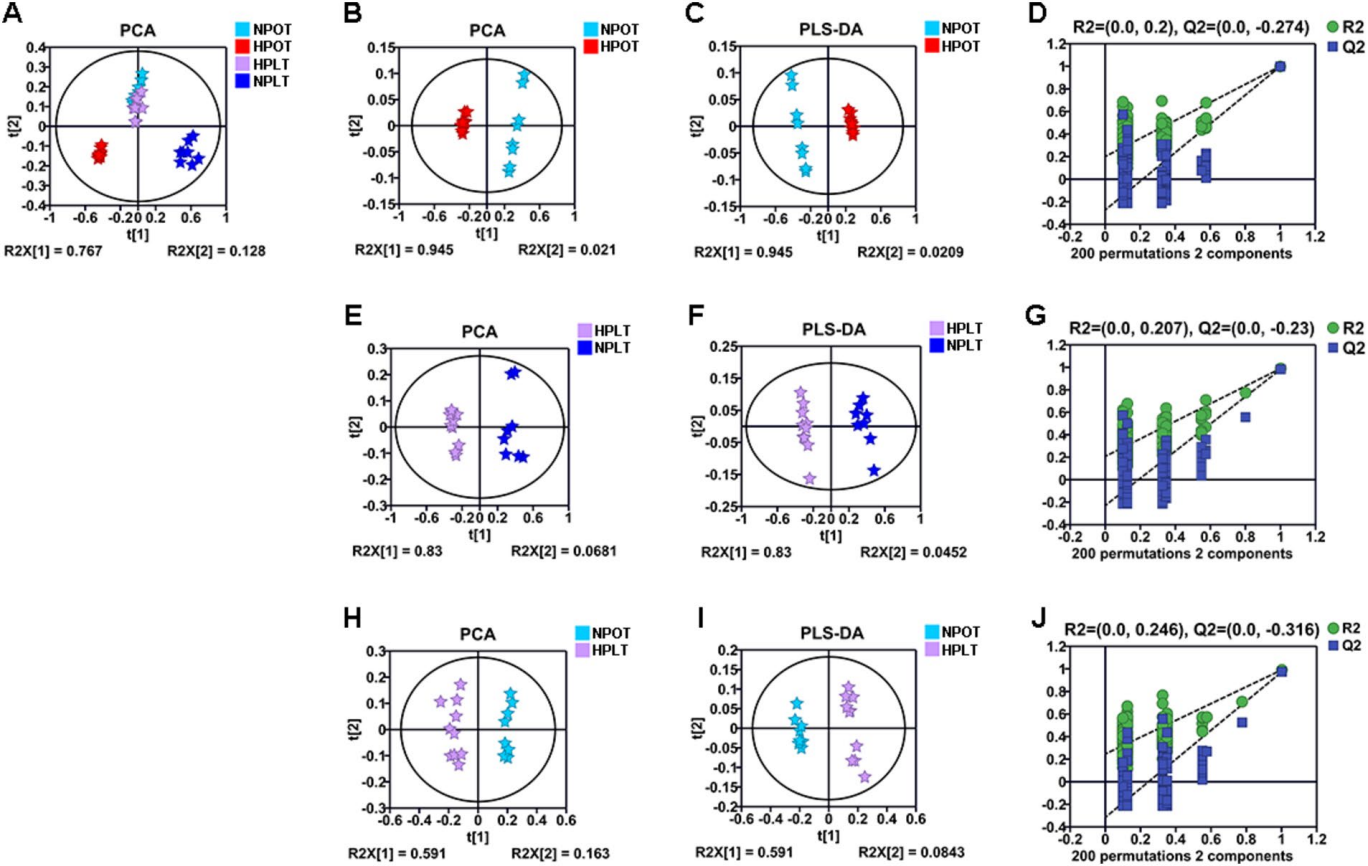
Multivariate statistical analyses for NMR data of the four groups of YLB-09 cells. (A) Scores plots of PCA models of four groups. (B–D) Scores plots of PCA and PLS-DA models of HPOT vs. NPOT, and cross-validation plot of the PLS-DA model. (E–G) Scores plots of PCA and PLS-DA models of HPLT vs. NPLT, and cross-validation plot of the PLS-DA model. (H–J) Scores plots of PCA and PLS-DA models of HPLT vs. NPOT, and cross-validation plot of the PLS-DA model.

Multivariate statistical analysis of the metabolite concentrations in the different treatment groups demonstrated that both high pressure and low temperature significantly altered the metabolic pattern of YLB-09 (Figure 2). There were significant differences in the metabolic pattern when high pressure was applied alone (HPOT vs. NPOT) and when high pressure was accompanied by low temperature (HPLT vs. NPLT), indicating that YLB-09 metabolism was strongly impacted by high pressure. Pairwise comparisons of the experimental groups under the influence of high pressure yielded the following values for R^2^X, R^2^Y, and Q^2^: R^2^X = 0.966, R^2^Y = 0.999, and Q^2^ = 0.998 for HPOT vs. NPOT (Figures 2B–D); R^2^X = 0.875, R^2^Y = 0.989, and Q^2^ = 0.981 for HPLT vs. NPLT (Figures 2E–G); and R^2^X = 0.675, R^2^Y = 0.990, and Q^2^ = 0.971 for HPLT vs. NPOT (Figures 2H–J). Collectively, these findings indicated the significant impact of high pressure on YLB-09. A similar comparison of the effect of low temperature on YLB-09 metabolism showed a clear differentiation in the metabolic patterns between low temperature (with or without high pressure) and optimal temperature conditions (Supplementary Figure S3).

### Screening of differential metabolites

Differential metabolites were identified by comparing the trends in metabolite concentrations between the groups, with a significance level set at *p* < 0.05 (Table 1; Supplementary Table S2). In total, 33 differential metabolites were identified under high-pressure conditions (HPOT vs. NPOT) and 28 were identified under high-pressure conditions accompanied by low temperature (HPLT vs. NPLT). The classification and number of differential metabolites induced by high-pressure conditions included the following: 13 metabolites related to the metabolism of amino acids (e.g., threonine, alanine, valine, and serine); six related to the metabolism of carbohydrates and their derivatives (e.g., citrate, lactate, and succinate); five associated with nucleotide metabolism (e.g., thymidine and uracil); and nine linked to other metabolic processes (e.g., ethanolamine, propylene glycol, and glycerol).

**Table 1:** Quantitative comparisons of metabolite concentrations between the four groups of YLB-09 cells under high pressure (with or without low temperature).

Metabolites	Mean ± SD				Significance		
	NPOT	HPOT	NPLT	HPLT	HPOT vs. NPOT	HPLT vs. NPLT	HPLT vs. NPOT
Amino acid							
Alanine	21.795 ± 0.679	11.315 ± 0.113	25.192 ± 1.503	18.964 ± 0.811	****	****	****
Aspartate	5.213 ± 0.270	3.034 ± 0.251	9.622 ± 0.341	6.563 ± 0.833	****	****	***
Glutamate	28.045 ± 0.870	16.246 ± 0.411	46.649 ± 6.653	28.544 ± 1.932	****	****	ns
Glycine	12.191 ± 0.237	7.636 ± 0.334	14.541 ± 0.686	13.508 ± 1.194	****	*	**
Histidine	1.894 ± 0.474	0.446 ± 0.182	0.482 ± 0.108	0.765 ± 0.201	****	**	***
Isoleucine	11.620 ± 0.316	6.364 ± 0.258	12.579 ± 0.666	10.716 ± 0.500	****	****	***
Leucine	16.372 ± 0.394	8.414 ± 0.168	17.122 ± 0.790	14.754 ± 0.758	****	****	****
Phenylalanine	9.096 ± 0.792	5.943 ± 0.291	6.572 ± 0.688	6.551 ± 1.058	****	ns	****
Serine	10.368 ± 2.180	4.535 ± 0.481	15.306 ± 1.672	13.603 ± 2.238	***	ns	**
Threitol	49.090 ± 1.058	34.057 ± 0.704	97.887 ± 6.132	60.805 ± 3.715	****	****	****
Threonine	39.128 ± 1.180	22.298 ± 0.541	66.335 ± 5.013	43.123 ± 2.032	****	****	***
Tyrosine	2.356 ± 0.779	1.137 ± 0.189	2.796 ± 0.289	1.433 ± 0.681	**	****	*
Valine	13.703 ± 0.285	8.103 ± 0.321	15.178 ± 0.838	12.811 ± 0.506	****	****	***
Carbohydrate							
Acetate	10.188 ± 0.306	9.780 ± 0.119	9.135 ± 0.211	11.176 ± 1.196	**	***	*
Betaine	1.005 ± 0.126	0.618 ± 0.037	3.378 ± 0.133	1.741 ± 0.162	****	****	****
Citrate	38.406 ± 0.888	22.661 ± 0.266	38.043 ± 1.811	32.682 ± 2.527	****	***	****
Formate	3.539 ± 0.396	3.926 ± 0.290	6.574 ± 0.851	4.980 ± 0.675	*	***	****
Glucose	0.053 ± 0.124	0.040 ± 0.060	9.769 ± 1.089	0.012 ± 0.037	ns	****	ns
Lactate	36.409 ± 2.740	26.127 ± 1.787	36.131 ± 4.770	41.923 ± 4.524	****	*	**
Succinate	1.371 ± 0.108	0.982 ± 0.052	1.629 ± 0.061	2.254 ± 0.343	****	***	****
Nucleotide							
1,7-Dimethylxanthine	0.938 ± 0.462	0.163 ± 0.126	0.679 ± 0.151	0.190 ± 0.154	**	****	**
Oxypurinol	2.415 ± 0.710	0.363 ± 0.295	1.865 ± 0.415	0.590 ± 0.342	****	****	****
Thymidine	8.327 ± 0.266	4.855 ± 0.106	11.078 ± 0.534	8.207 ± 0.493	****	****	ns
Uracil	6.350 ± 1.560	1.219 ± 0.235	6.483 ± 0.482	3.266 ± 0.938	****	****	****
Xanthine	3.622 ± 0.823	0.586 ± 0.272	1.048 ± 0.432	1.135 ± 0.519	****	ns	****
Others							
Creatine	2.096 ± 0.178	0.968 ± 0.072	3.471 ± 0.183	2.161 ± 0.425	****	****	ns
Ethanol	39.856 ± 3.523	29.289 ± 1.858	35.142 ± 1.652	37.870 ± 2.119	****	**	ns
Ethanolamine	43.413 ± 0.606	14.183 ± 0.492	74.594 ± 3.424	26.797 ± 1.603	****	****	****
Glycerol	64.294 ± 2.121	59.948 ± 0.839	83.131 ± 4.057	71.841 ± 4.908	****	****	***
Imidazole	1.370 ± 0.442	0.231 ± 0.124	1.577 ± 0.544	0.671 ± 0.260	***	***	***
Methanol	3.802 ± 0.898	2.781 ± 0.733	46.521 ± 5.264	4.118 ± 0.558	*	****	ns
Propylene glycol	12.967 ± 0.712	9.819 ± 0.452	12.280 ± 0.931	12.594 ± 0.649	****	ns	ns
Pyridoxine	1.351 ± 0.582	0.282 ± 0.219	0.546 ± 0.234	0.818 ± 1.146	***	ns	ns
TMAO	5.215 ± 0.216	2.844 ± 0.052	5.794 ± 2.889	4.893 ± 1.638	****	ns	ns

Statistical significances of differences in metabolite concentration were analyzed by independent sample *t*-test: ns, *p* > 0.05; *, *p* < 0.05; **, *p* < 0.01; ***, *p* < 0.001; ****, *p* < 0.0001. Red, increase in metabolite concentration; blue, decreases in metabolite concentration.

### Screening for characteristic metabolites

**Figure 3:**
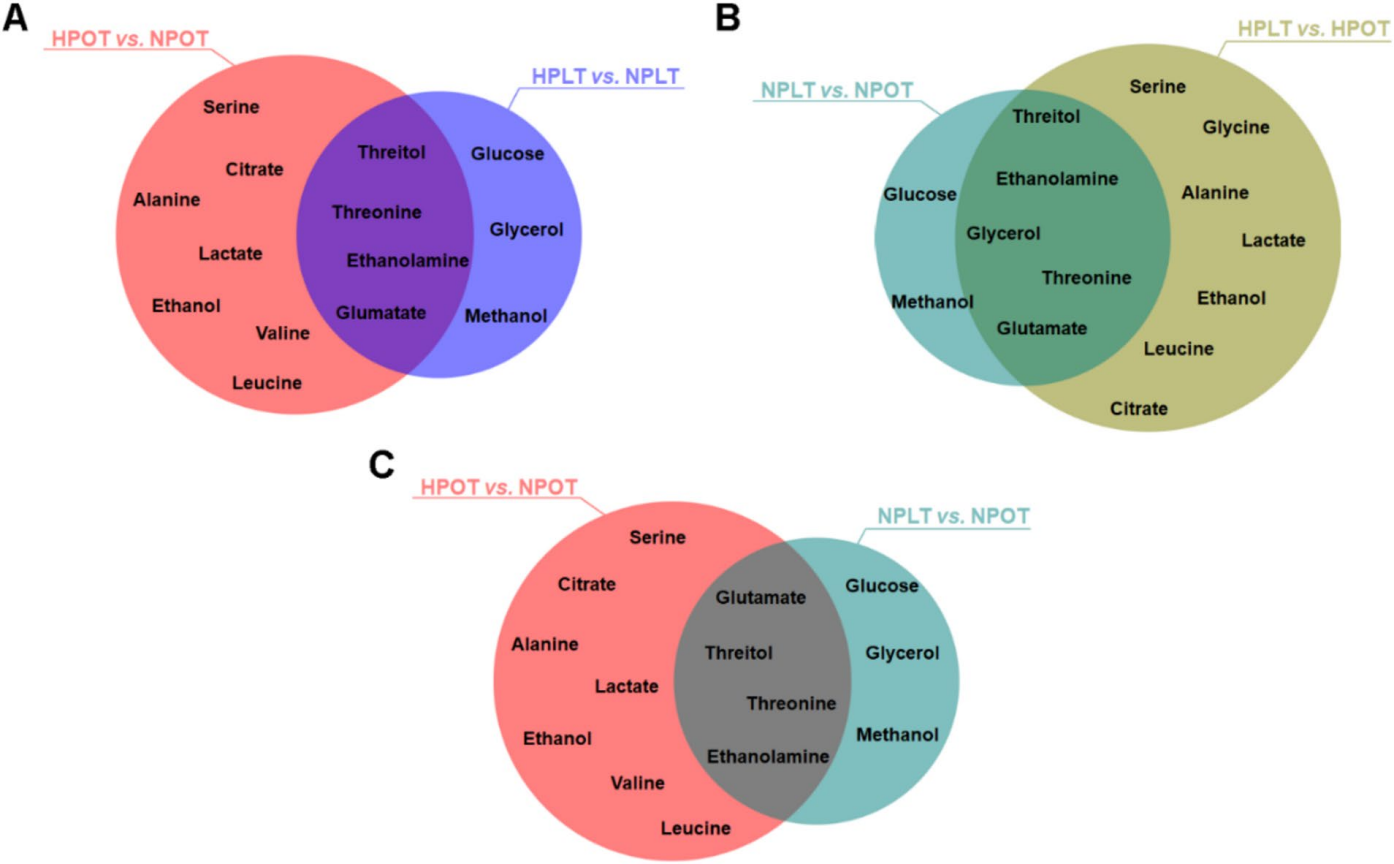
Venn plots of characteristic metabolites for two-by-two comparisons of experimental groups. The intersections of the characteristic metabolites that screened during the two-by-two comparisons of the experimental groups were identified and represented using Venn diagrams.

Characteristic metabolites were identified by employing cutoffs of VIP >1 and *p* < 0.05. The comparison between the HPOT and NPOT groups revealed 11 distinct characteristic metabolites, while the comparison between HPLT and NPLT yielded seven. Furthermore, analyses of characteristic metabolites under low-temperature conditions identified seven characteristic metabolites in the NPLT vs. NPOT comparison and 12 in the HPLT vs. HPOT comparison. A search for metabolites common to all four groups revealed several that may be associated with the high-pressure adaptation of strain YLB-09, namely ethanolamine, citrate, lactate, alanine, valine, serine, threonine, glutamate, and threitol (Figure 3).

### Metabolic pathway enrichment analysis

**Figure 4:**
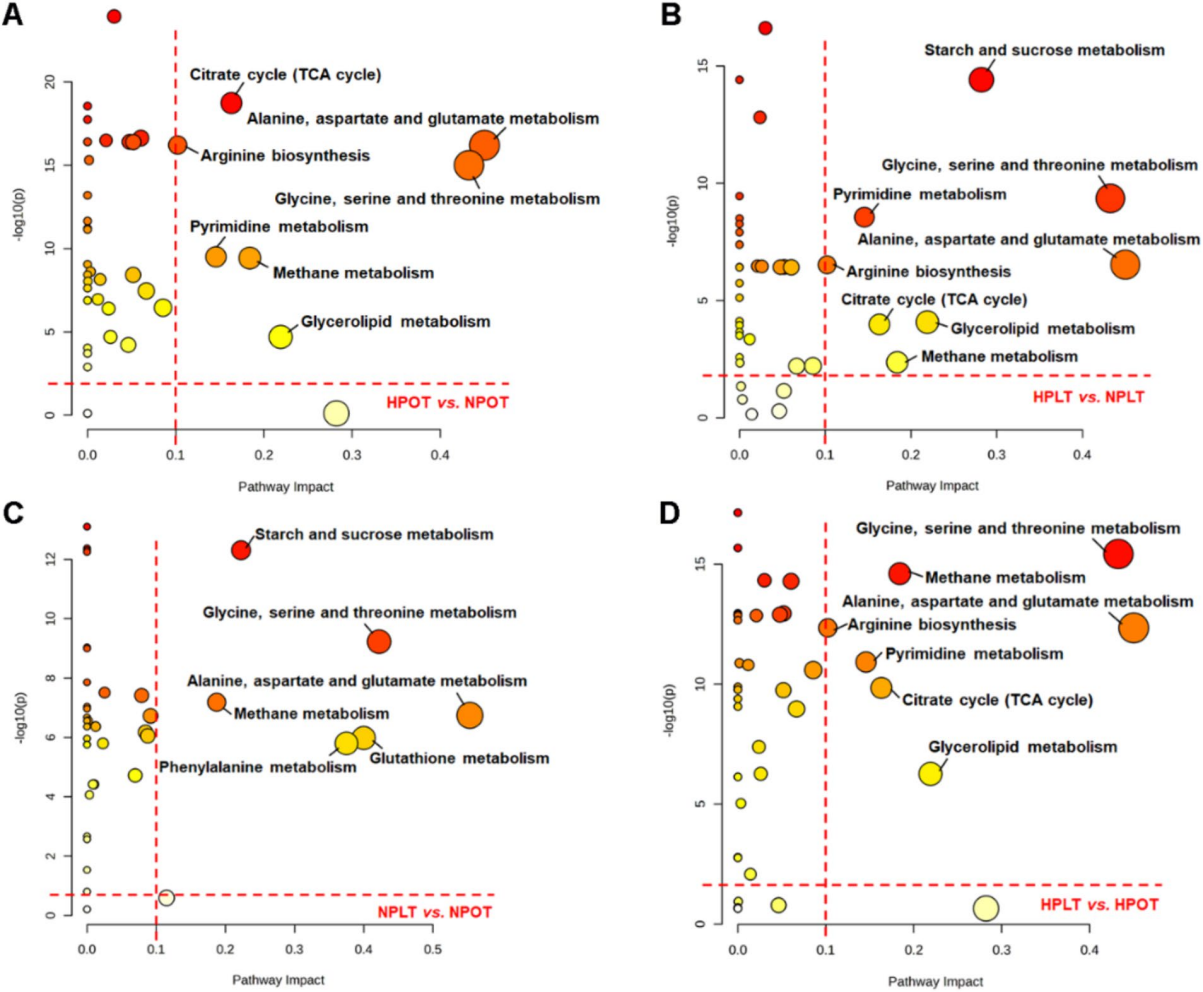
Metabolic pathway analysis conducted on YLB-09 cells exposed to high-pressure and low temperature conditions. Significantly altered metabolic pathways were identified with pathway impact value (PIV) >0.1 and *p* < 0.05, using the pathway analysis module available on the MetaboAnalyst 5.0 webserver. (A) HPOT vs. NPOT, (B) HPLT vs. NPLT, (C) NPLT vs. NPOT, (D) HPLT vs. HPOT.

The metabolic pathways that exhibited significant regulation between groups were subjected to enrichment analysis, utilizing data on metabolite nomenclature and associated concentration alterations. This analysis identified eight metabolic pathways with a significant regulatory change under high-pressure conditions, regardless of the temperature (Figure 4; Supplementary Figure S4). The identified pathways were found to be related to energy metabolism [e.g., tricarboxylic acid (TCA) cycle and sucrose metabolism]; amino acid metabolism (e.g., alanine metabolism, glycine metabolism, and arginine metabolism); and glycerolipid metabolism. Similarly, the pathways that were significantly altered by low-temperature conditions were the same as those altered by high-pressure conditions. This finding suggested that the effects of high pressure on the molecular and biological processes of strain YLB-09 may be similar to those of low temperature.

### Annotation of DEGs in the transcriptome and association analysis with the metabolome

**Figure 5:**
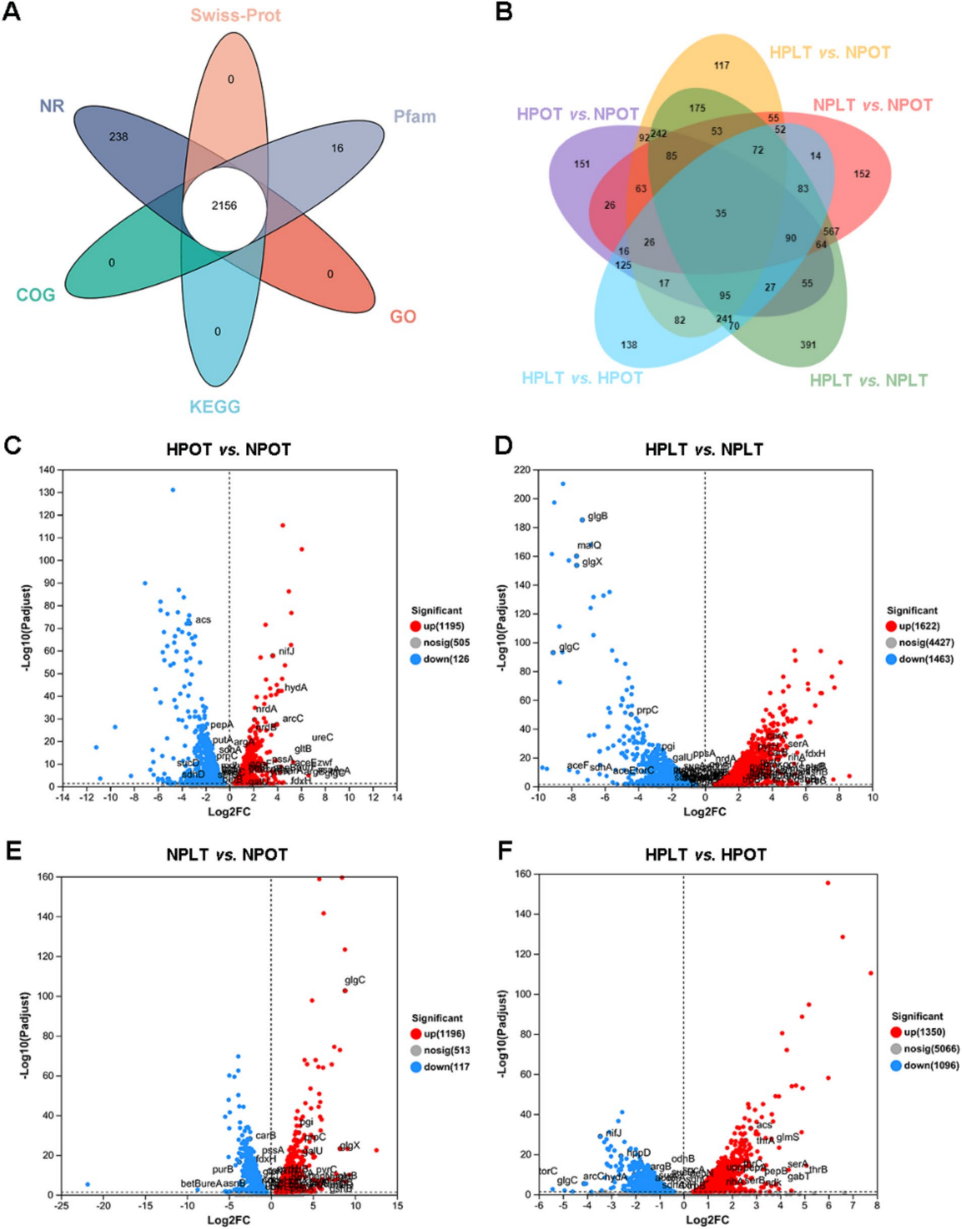
Venn plots of gene annotation of transcriptome data. (A) Annotation of coding genes in 5 major databases. (B) Overlap of differentially expressed genes in two-by-two comparisons of experimental groups. (C) Volcano plot of DEGs in HPOT vs. NPOT. (D) Volcano plot of DEGs in HPLT vs. NPLT. (E) Volcano plot of DEGs in NPLT vs. NPOT. (F) Volcano plot of DEGs in HPLT vs. HPOT.

Annotation of the transcriptome data produced 2,969 coding genes co-annotated to five major databases (Figure 5A). Most of the total coding genes (5,087, 98.32%,) were annotated to the NR database. To assess the extent of alteration in gene expression between the groups, the DEGs were evaluated using a significance threshold of adjusted *p* < 0.05 (Figure 5B).

Next, we analyzed the metabolic pathways associated with the DEGs and compared them with those that were significantly regulated in the annotated metabolomics data. Some of the pathways annotated in the metabolomics analysis featured DEGs whose expression trends coincided with the direction of pathway regulation, which we labeled on volcano maps of the DEGs (Figures 5C–F).

**Figure 6:**
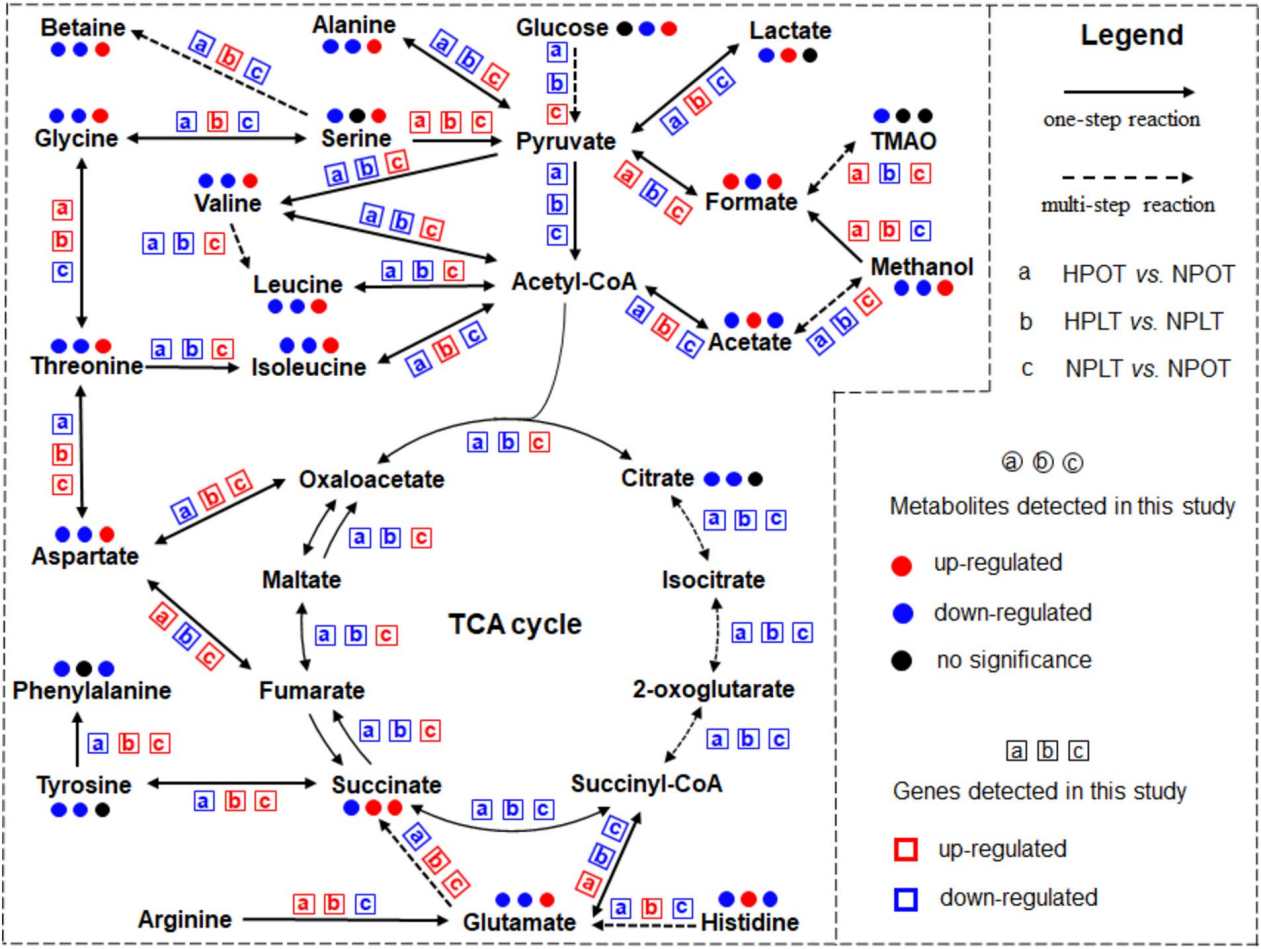
Significant metabolic pathways identified from the analysis under high pressure and low temperature conditions. Metabolites identified by a comprehensive analysis combining metabolomics and transcriptomics approaches are included in the highlighted pathways. The change trends in metabolite concentrations and gene expressions are shown for further insight.

In addition, we mapped an overview of some of the metabolites that corresponded to the metabolic pathways (Figure 6). To better visualize the regulatory effects of the DEGs on the above-mentioned metabolic pathways, we also examined changes in the expression of the genes corresponding to the metabolic processes. The results showed that the trend of regulated gene expression under both high-pressure and low-temperature conditions was consistent with the corresponding changes in metabolite concentrations.

### Associations of characteristic metabolites and DEGs with significantly regulated pathways

An enrichment analysis of the DEGs using the KEGG database revealed that some of the top 20 most enriched functional gene sets/metabolic pathways under the high-pressure environment had significant impacts on life-sustaining processes of YLB-09. These included respiratory chain and energy metabolism, carbohydrate and its intermediates metabolism, amino acid metabolism, ion/substance transport, and biofilm production (Supplementary Figure S5). This finding suggested that the regulation of these processes may be closely related to the adaptation of YLB-09 to the high-pressure environment of the deep sea.

**Figure 7:**
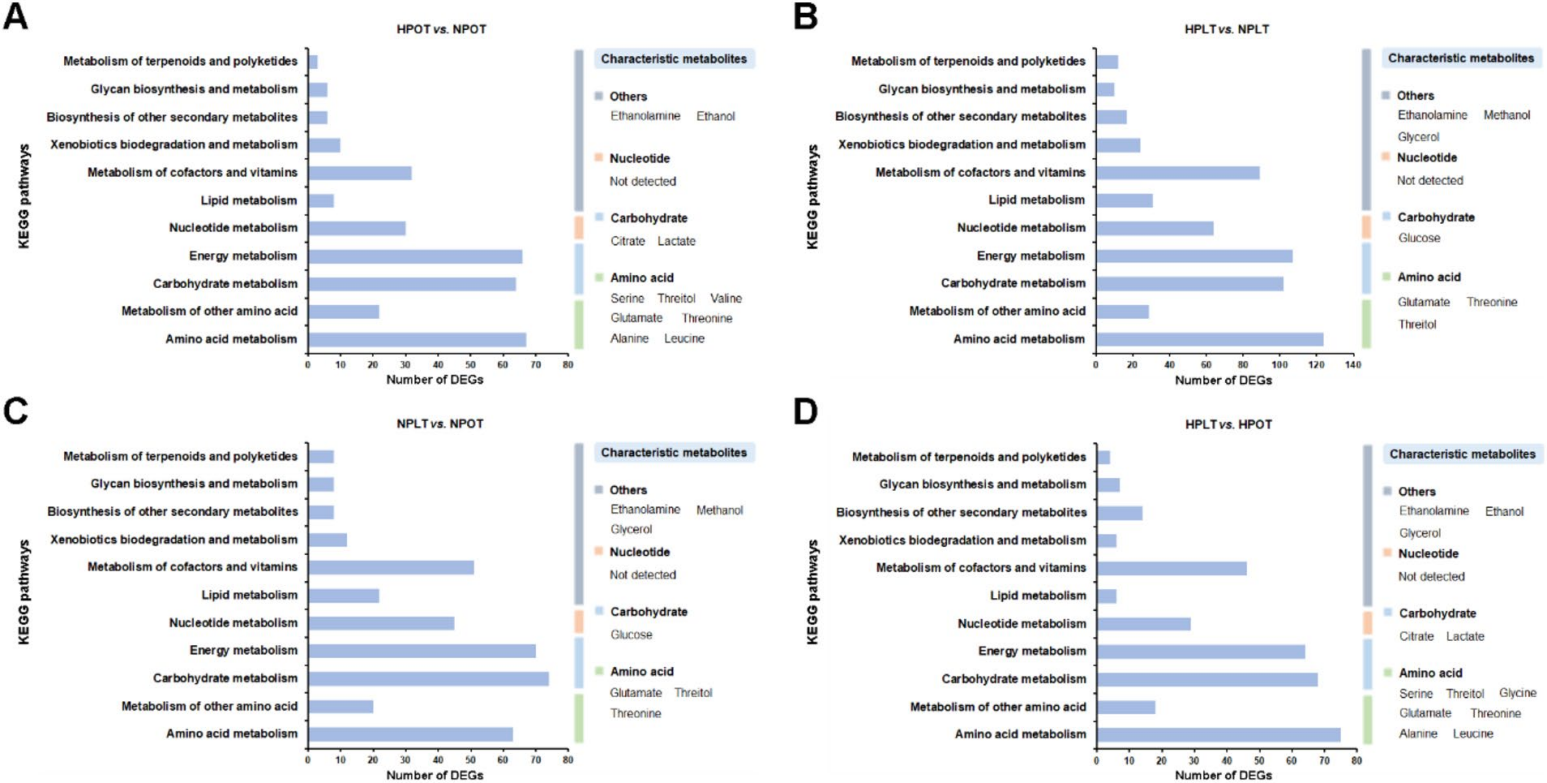
Association analysis of the KEGG enrichment results of the DEGs with the characteristic metabolites. (A) HPOT vs. NPOT, (B) HPLT vs. NPLT, (C) NPLT vs. NPOT, (D) HPLT vs. HPOT.

In addition, we correlated the KEGG enrichment results for the top 20 DEGs related to metabolic processes with the metabolites characterized in this study (Supplementary Table S3). The results confirmed that the metabolic processes affected by high-pressure and low-temperature conditions were similar. These processes were enriched in energy metabolism (including carbohydrate metabolism), amino acid metabolism, and other processes, including lipid metabolism. Accordingly, under high-pressure conditions (HPOT vs. NPOT), the characterized metabolites serine, threitol, valine, glutamate, threonine, alanine, and leucine corresponded to pathways involving amino acid metabolism, citrate and lactate to carbohydrate metabolism, and ethanolamine and ethanol to other metabolic pathways (Figure 7A). Similar results were obtained for the other paired groups (Figures 7B–D).

## Discussion

### *Shewanella eurypsychrophilus* YLB-09 adapts to high-pressure environments by switching from aerobic oxidation to trimethylamine N-oxide (TMAO) respiration

The effects of pressure on deep-sea bacteria have been increasingly elucidated, with a growing body of evidence indicating that pathways involving amino acid metabolism, solute transport, and energy metabolism are primarily affected (Vannier et al., 2015; Amrani et al., 2014). We identified a total of six energy-related metabolites in our NMR-based metabolomic analysis, namely acetate, betaine, citrate, formate, lactate, and succinate. Furthermore, two distinctive metabolites, lactate and citrate, were found to differentiate the high-pressure and normal-pressure metabolic patterns. A notable regulation of the TCA cycle in relation to energy metabolism was also identified through the metabolic pathway enrichment analysis. Our findings suggested that the high-pressure acclimatization of strain YLB-09 may be associated with the regulation of energy metabolism. Others have observed that microorganisms adapted to extreme environments reduce their metabolic rate through processes such as intracellular energy metabolism. Additionally, the intracellular ATP content of certain pathogenic bacteria has been shown to gradually decline in response to increased environmental pressure or prolonged pressurization (Tholozan et al., 2000; Ukuku et al., 2009). Our group previously used NMR to examine the metabolomic data of *Microbacterium sediminis* strain YLB-01 under high-pressure conditions. The findings of that study indicated that intracellular energy metabolism was significantly regulated under high-pressure conditions (Qiu et al., 2024), aligning with the pronounced disturbance in energy metabolism in response to elevated pressure in strain YLB-01 in the current investigation. It is evident that the regulation of energy metabolism plays an indispensable role in the adaptation of deep-sea microorganisms to high pressure levels.

To validate this assertion, we conducted an additional data mining exercise using the transcriptome of YLB-09 under elevated pressure conditions (Supplementary Table S4). Consistently, KEGG annotation analysis of the transcriptomic data indicated significant disruption of metabolic pathways involved in intracellular energy production of YLB-09 (Supplementary Figure S6). Specifically, high-pressure conditions in YLB-09 cells caused widespread downregulated expression of genes involved in three pathways (TCA cycle, pyruvate metabolism, and oxidative phosphorylation), corresponding to decreased concentrations of the metabolites citrate, succinate, and acetate (Supplementary Figures S6A–C). In contrast, genes involved in the glycolysis/gluconeogenesis pathway were generally upregulated (Supplementary Figure S6D). Taken together, these findings suggested that YLB-09 may have a tendency to switch from aerobic respiration for energy production to anaerobic respiration under high-pressure conditions. A number of studies have reported that strains of the genus *Shewanella* adapt to extreme environments by performing anaerobic respiration (Wang et al., 2008). Li et al. (2018) found that *Shewanella piezotolans* WP3 was capable of nitrate respiration at high hydrostatic pressures, and that this process depended on its two periplasmic nitrate reductase (NAP) systems, involving the closely related enzymes NAP-α and NAP-β (Xiao et al., 2007). In their investigation of the mechanism of dimethylsulfoxide (DMSO) anaerobic respiration, Xiong and colleagues proposed that the DMSO anaerobic respiration exhibited by strain WP3 may be closely associated with its adaptation to the high-pressure environment typical of the deep sea (Xiong et al., 2016). Furthermore, cytochrome protein complexes C and D of a piezotolerant strain of *Shewanella violacea* exhibited differential regulation under high-pressure conditions (Tamegai et al., 2012).

Surprisingly, the previous genome annotation of strain YLB-09 revealed the presence of additional respiratory systems, allowing for anaerobic respiration through varied electron acceptors, including nitrate, TMAO, DMSO, singlet sulfur, and Fe^3+^ (Cao et al., 2022). To further clarify how YLB-09 achieves productive growth under high-pressure conditions, we analyzed the expression of the TMAO respiration-related *tor* genes under high-pressure conditions coupled with the previous functional annotation of YLB-09 genome (Supplementary Figures S7A–F). Surprisingly, we found significant upregulation of *torS*, *torR*, *torC*, *torA*, *torD*, and *torT*, resulting in upregulation of the TMAO respiratory system and simultaneous depletion of the TMAO substrate, in YLB-09 cells under high-pressure conditions (Supplementary Figure S7G). However, this upregulation of TMAO respiration was not observed under low-temperature conditions (Supplementary Figure S7H). It has been observed that a considerable number of piezotolerant bacteria are able to adapt to high-pressure environments by modifying their respiratory chain (Oger and Jebbar, 2010). The metabolomic and transcriptomic findings of this study indicated that YLB-09 may regulate energy metabolism through multiple anaerobic respiratory systems, such as TMAO respiration, which could also be a key mechanism through which it has adapted to the high-pressure environment of the deep sea.

### *Shewanella eurypsychrophilus* YLB-09 adapts to high-pressure environments by altering amino acid metabolic profiles

Our metabolic pathway enrichment analysis indicated that high pressure exerted a notable influence on a range of amino acid metabolic processes in YLB-09, including alanine, aspartate, and glutamate metabolism; arginine metabolism; and glycine, serine, and threonine metabolism. Additionally, KEGG analysis at the level of gene transcription revealed that high pressure significantly impacted the amino acid metabolism of YLB-09. Amino acid metabolism is intimately associated with protein synthesis and degradation in bacteria. Ohmae et al. (2018) observed that the 3-isopropylmalate dehydrogenase (IDH) enzymes of *Shewanella benthica* strains DB21MT-2 and MR-1 exhibited different pressure sensitivities: IDH from DB21MT-2 was pressure tolerant up to 100 MPa, whereas IDH from MR-1 was pressure sensitive. This difference was attributed to the coding of serine versus alanine at amino acid residue 266 of the enzyme. If we posit that amino acid changes in proteins can affect pressure adaptation in bacterial strains, it follows that the regulation of amino acid metabolism within strains may be related to pressure tolerance.

It has been demonstrated that cryotolerant bacteria respond to low temperatures by undergoing changes in amino acid composition (Giordano et al., 2012). In the bacterial genus *Shewanella*, the proteomes of cold-tolerant species exhibit reduced levels of alanine, proline, glycine, and arginine, along with elevated levels of lysine, isoleucine, and asparagine compared with those of warm-adapted species (Zhao et al., 2010). This suggests a potential correlation between an amino acid profile and the structural flexibility of a protein under extreme conditions. Similarly, it can be posited that extreme conditions affecting strain growth may also influence the ability of the strain to adapt to pressure via alterations to its amino acid profile. Some studies have pointed out that piezotolerant strains of the genera *Colwellia*, *Psychromonas*, and *Shewanella* possess high proportions of basic proteins, which may serve to maintain a charge balance in the cytoplasm (Peoples et al., 2020). Additionally, high abundances of tryptophan, tyrosine, leucine, phenylalanine, and methionine were observed in the piezophilic strains, in contrast to high abundances of glutamate, aspartate, asparagine, and serine displayed in the pressure-sensitive strains (Peoples et al., 2020). The metabolomic results of the current study indicated that high-pressure treatment of strain YLB-09 not only reduced the intracellular concentrations of glutamate and serine, it also diminished the concentrations of other amino acids, consistent with the observations reported by Nguyen et al. (2019). This alteration may be associated with modifications to the amino acid profile, as well as shifts in the rates of protein synthesis and degradation, under high-pressure conditions.

Further analysis of the transcriptomic data for the expression of genes related to amino acid metabolism under high-pressure conditions is provided in Supplementary Table S5. Consistent with the above results, the amino acid metabolism of YLB-09 was generally downregulated under high pressure, with corresponding trends in the expression of related genes and substrate/product concentrations. In summary, our findings suggested that YLB-09 may adapt to the high-pressure environment by modulating its amino acid metabolic profile.

### *Shewanella eurypsychrophilus* YLB-09 adapts to high-pressure environments by altering the composition and the fluidity of the cell membrane

The metabolite analysis revealed a notable reduction in the ethanolamine concentration in YLB-09 when exposed to high-pressure conditions. Furthermore, ethanolamine was found to be a characteristic metabolite for the differentiation of the metabolic profiles of YLB-09 under high-pressure and normal-pressure conditions. Ethanolamine is involved in the synthesis of phosphatidylethanolamine, which is the most abundant lipid in the cell membranes of Gram-negative bacteria. This lipid acts as a signaling molecule to regulate cell metabolism (Rothfield and Romeo, 1971; Dowhan, 2009). The observed changes in ethanolamine concentration suggested that high pressure can affect the cell membrane composition of strain YLB-09.

Among the harsh conditions of the deep sea, hydrostatic pressure reportedly has the strongest influence on the cell membrane composition of bacteria (Michoud and Jebbar, 2016). The cell membrane has profound impacts on a range of processes, including the facilitation of nutrient and ion transport and solute uptake, and the regulation of osmotic pressure (Strahl and Errington, 2017). The loss of cellular membrane integrity resulting from high pressure is accompanied by leakage of ATP, proteins, and other crucial components, in addition to the disappearance of certain outer membrane proteins (Pagan and Mackey, 2000; Ritz et al., 2000). Concurrently, the dissolution of the cell membrane during the exponential growth phase renders bacterial cells non-viable (Pagan and Mackey, 2000; Ulmer et al., 2000). Therefore, regulation of cell membrane composition represents a crucial strategy by which microorganisms can cope with environments characterized by high pressure levels. Our results demonstrated that the intracellular ethanolamine concentration of YLB-09 was significantly reduced during high-pressure treatment. Furthermore, KEGG annotation of gene transcripts indicated that high-pressure conditions affected cell membrane synthesis-related processes. Consequently, our findings indicated that the components of the YLB-09 cell membrane are significantly regulated under high-pressure conditions.

Furthermore, metabolic pathway enrichment analysis indicated that the glycerolipid metabolism pathway exhibited significant regulatory alterations in response to high-pressure conditions. Wang et al. (2014) observed this phenomenon in their investigation of *Sporosarcina* sp. DSK25, a pressure-tolerant bacterium. They reported a notable increase in the concentration of polyunsaturated fatty acids (PUFAs) in the strain as pressure levels increased. Additionally, the PUFA composition of the membrane lipids has been shown to play a pivotal role in determining the resilience of piezophilic/piezotolerant bacteria to elevated pressure (Usui et al., 2012). The composition of fatty acids in the cell membrane is a crucial determinant of membrane fluidity. Glycerolipid metabolism is intricately linked to cell membrane fatty acid metabolism, which in turn exerts a profound influence on cell membrane fluidity. Increased pressure levels have also been observed to result in a reduction in cell membrane fluidity (Macdonald et al., 1988). Uniquely, we also annotated DEGs related to fatty acid metabolism. In particular, some genes related to fatty acid biosynthesis were upregulated and some were downregulated, and the same was true for genes related to fatty acid degradation (Supplementary Table S6). This suggested that YLB-09 regulates the cell membrane characteristics affected by the lipid composition, such as cell membrane fluidity, by modulating fatty acid metabolism.

Proper fluidity of biological membranes is essential for the maintenance of membrane structure and function. Casadei et al. (2002) observed that elevated pressure may result in alterations to the lipid composition of bacterial cell membranes, leading to tighter packing of the phospholipid bilayer acyl chains. Additionally, the pressure tolerance of cells in logarithmic growth phase is closely associated with their membrane fluidity. Some pressure-sensitive bacteria also exhibit changes in the amount and proportion of monounsaturated fatty acids in their cell membranes in response to high hydrostatic pressure (Delong and Yayanos, 1986). As evidenced by our findings, YLB-09 exhibits regulation of fatty acid metabolism and cell membrane composition under conditions of elevated pressure. The consequent alterations in the fluidity and composition of the cell membranes enable YLB-09 to adapt to the high-pressure environment.

### *Shewanella eurypsychrophilus* YLB-09 responds differently to high pressure and low temperature

To better understand the distinct survival strategies of YLB-09 under high-pressure and low-temperature conditions, we carried out a comparative analysis of the respective metabolic and gene transcription profiles. Specifically, we focused on three areas: energy production, amino acid metabolism, and cell membrane synthesis.

Regarding energy generation, we found that, under high-pressure conditions, YLB-09 exhibited reduced aerobic energy metabolism and enhanced anaerobic respiration, namely TMAO respiration. By contrast, at low temperatures, aerobic metabolism was preserved in YLB-09, mainly evidenced by upregulation of TCA cycle substrate concentrations and genes (Supplementary Figure S8 and Supplementary Table S2) and upregulated expression of oxidative phosphorylation genes (Supplementary Figure S8). In our previous exploration of the cold-adapted metabolic profile of psychrotolerant strain *M. sediminis* YLB-01, inconsistent with the results of the present study, we detected enhanced glycolysis and increased levels of related metabolic products at low temperatures, but no significant change in lactate concentration at low temperatures. This apparent discrepancy in energy metabolism profiles at low temperatures may be related to the clear preference of psychrotrophic strain YLB-09 for low temperatures (unable to grow at temperatures above 20°C) compared with that of the psychrotolerant strain YLB-01 (Xia et al., 2020). Furthermore, our previous study also found that low temperature had a significantly greater impact on bacterial growth than high pressure (Qiu et al., 2024). However, in this study, we observed that, even at low temperature, the effects of high pressure on the production mode of YLB-09 remained unchanged. This may represent a unique strategy that psychrotrophic bacteria have evolved to cope with low temperatures during their long-term adaptation to the extreme environment of the deep sea.

Regarding amino acid metabolism, we observed that low-temperature conditions induced the accumulation of amino acids in YLB-09, including upregulated levels of glutamate, threonine, and valine, in contrast to the general downregulation of amino acid metabolism under high-pressure conditions (Supplementary Table S2). Indeed, psychrotrophic bacteria have been shown to contain significantly more glutamate, valine, alanine, aspartate, and threonine, and less leucine and lysine, than their cold-intolerant counterparts (Metpally and Reddy, 2009; Dsouza et al., 2015; Aliyu et al., 2016; Dethlefsen et al., 2019). Thus, YLB-09 has adapted to the low-temperature environment by modulating its metabolic profile of amino acids. Additionally, in metabolomics studies of the strain *Mesorhizobium* sp. N33, the compatible solutes threonine and valine were found to be accumulated under low-temperature conditions (Ghobakhlou et al., 2013). Consistently, our metabolomics analysis also identified threonine as a characteristic metabolite of YLB-09 in response to low temperature as well as high pressure. This finding suggested that YLB-09 may be able to adapt to the low-temperature environment by increasing the production of threonine as a compatible solute.

Cell membrane synthesis under high-pressure conditions was marked by a significant reduction in ethanolamine concentration, with upregulation of some genes related to fatty acid metabolism, thereby regulating cell membrane fluidity. In contrast, under low-temperature conditions, YLB-09 exhibited ethanolamine accumulation, consistent with significant upregulation of genes related to fatty acid synthesis (Supplementary Figure S8 and Supplementary Table S6). It has been reported that *Yersinia pseudotuberculosis* significantly increases the lysophosphatidylethanolamine content, phase transition temperature, and hardness of the cell membrane under low-temperature stress (Li et al., 2004). YLB-09 may regulate cell membrane synthesis by accumulating ethanolamine, thus modulating membrane properties such as hardness to adapt to the low-temperature environment. Furthermore, studies on cold-tolerant marine bacteria have shown that the cell membranes of these bacteria contain large amounts of PUFAs (Wirsen et al., 1986). For example, Kawamoto et al. (2009) demonstrated that eicosapentaenoic acid plays an important role in cytokinesis and membrane biogenesis of *Shewanella livingstonensis* Ac10 at low temperature (Yokoyama et al., 2017). It has also been reported that some bacteria of the genus *Hivarius* are capable of producing PUFAs in response to low-temperature conditions (Ito et al., 2016). The upregulated expression of genes related to fatty acid biosynthesis revealed in our transcriptomic data may represent a mechanism by which YLB-09 regulates fatty acid metabolism to maintain the fluidity of the cell membrane at low temperatures.

## Conclusion

In this study, we investigated the metabolic regulatory mechanisms that enable the pressure-tolerant, cryophilic deep-sea bacterial strain *S. eurypsychrophilus* YLB-09 to adapt to high-pressure environments using NMR-based metabolomics and RNAseq-based transcriptomics analyses. Our findings highlight the pronounced impacts of high pressure levels on the metabolic profile of YLB-09, influencing the dynamics of energy metabolism, amino acid metabolism, and glycerolipid metabolism. Integration of the metabolomic and transcriptomic data revealed that YLB-09 may adapt to the high-pressure environment of the deep sea by regulating the following metabolic processes: (1) intracellular energy metabolism, through switching from aerobic respiration to TMAO respiration; (2) intracellular amino acid metabolism processes, altering the amino acid profile; and (3) fatty acid metabolism and synthesis at the cell membrane. The discovery of these pressure-adaptive mechanisms of strain YLB-09 provides a foundation for more detailed examinations of the survival mechanisms of life under high-pressure environments.

## Data Availability

The original contributions presented in the study are included in the article/supplementary material, further inquiries can be directed to the corresponding author.

## Footnotes

^1^ https://hmdb.ca/

^2^ http://deweylab.github.io/RSEM/

^3^ https://www.kegg.jp/kegg/mapper/color.html
