# Supplementary material for: Metabolic adaptations of Shewanella eurypsychrophilus YLB-09 for survival in the high-pressure environment of the deep sea
Source: Front Microbiol. 2024 Oct 17;15:1467153. doi: 10.3389/fmicb.2024.1467153 (PMC11527400; doi:10.3389/fmicb.2024.1467153)
Supplement: Supplementary file 1 [file Data_Sheet_1.zip › Data Sheet 1-20241014/Supplementary Materials-241014.DOCX]

**Supplementary Materials**

**Metabolic Adaptations of *Shewanella eurypsychrophilus* YLB-09 for Survival in the High-Pressure Environment of the Deep Sea**

**Journal:** Frontiers in Microbiology

**Authors:** Xu Qiu^1, 2^, Xixiang Tang^1,^ *

**Affiliations:**

1. State Key Laboratory Breeding Base of Marine Genetic Resources, Key Laboratory of Marine Genetic Resources, Fujian Key Laboratory of Marine Genetic Resources, Third Institute of Oceanography, Ministry of Natural Resources, 184 Daxue Road, Xiamen, China;
2. Key Laboratory for Chemical Biology of Fujian Province, MOE Key Laboratory of Spectrochemical Analysis and Instrumentation, College of Chemistry and Chemical Engineering, Xiamen University, Xiamen, China;

***Correspondence Authors:**

Xixiang Tang, E-mail address: [tangxixiang@tio.org.cn](mailto:tangxixiang@tio.org.cn).

**Figure S1.**


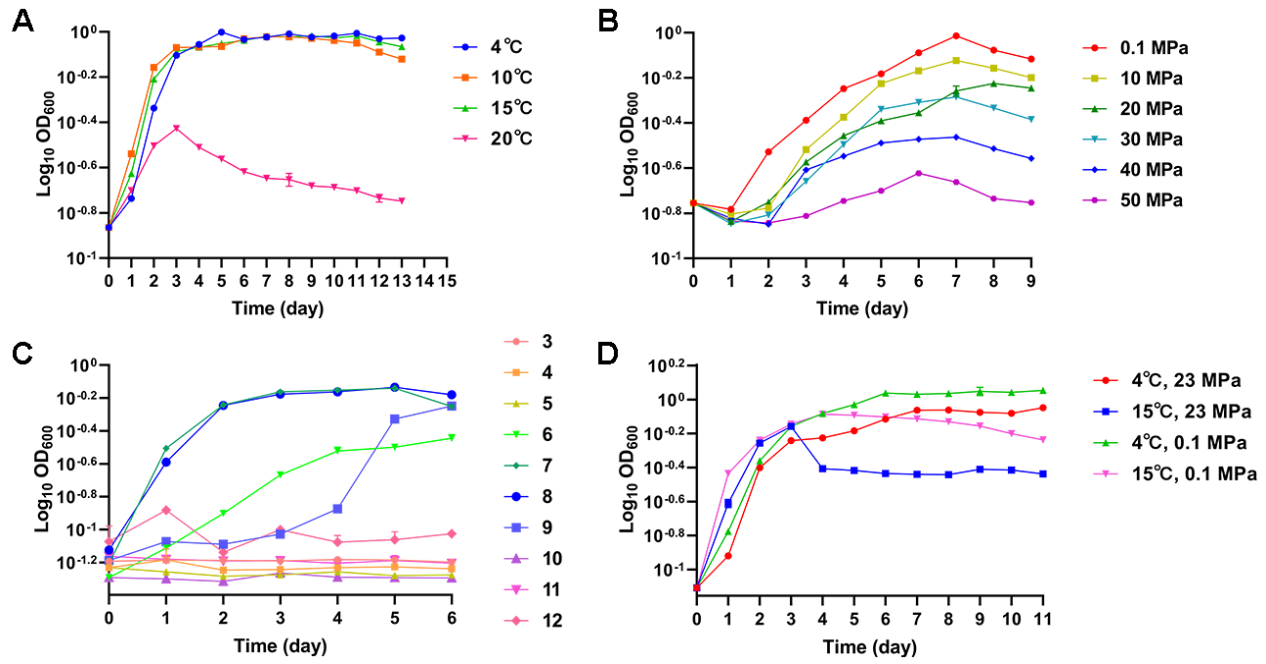


**Figure S1. Growth curves of YLB-09 cells at different temperatures (A), pressures (B), pH (C), and combinations of conditions (D).** To obtain the growth curves of YLB-01 under different conditions, the cultivation parameters of strain YLB-01 were as follows. (A) Growth curve under different temperature: Marine broth (2216E liquid medium), pH = 7.6, 0.1 MPa, temperature gradient of 4, 10, 15, 20, 25, 30°C, and in a shaker at 180 rpm. (B) Growth curve under different pressure: Marine broth (2216E liquid medium), 15°C, pH = 7.6, pressure gradient of 0.1, 10, 20, 30, 40, 50 MPa, and without shaking. (C) Growth curve under different pH: Marine broth (2216E liquid medium), 15°C, 0.1 MPa, pH gradient of 3, 4, 5, 6, 7, 8, 9, 10, 11, 12 and in a shaker at 180 rpm. (D) Growth curve under combined conditions: Marine broth (2216E liquid medium), pH = 7.6, based on the corresponding experimental conditions in an incubator protected from light and without shaking, at a temperature of 4 or 15 °C and a pressure of 0.1 or 23 MPa.

**Figure S2.**


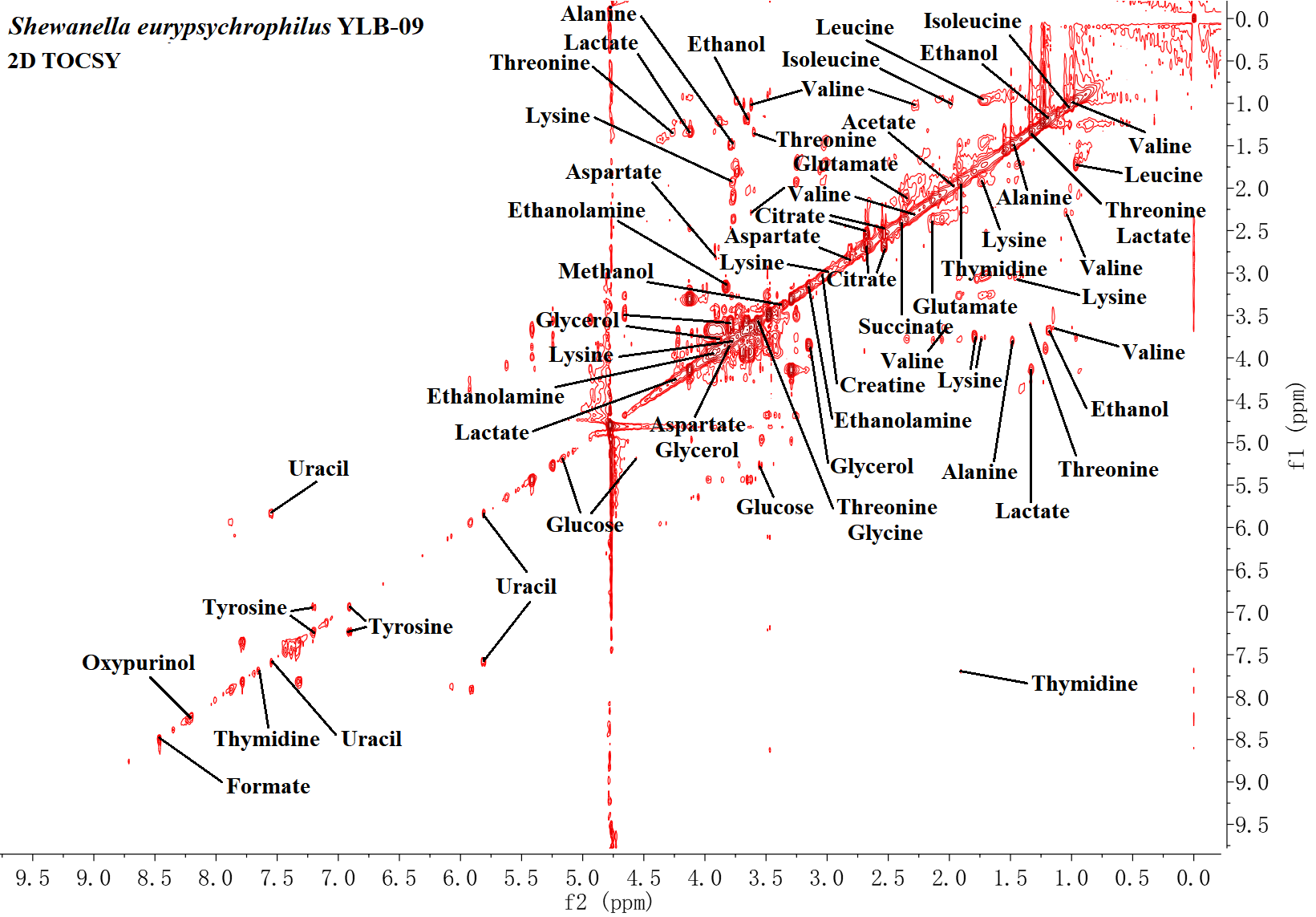


**Figure S2. Representative 2D ^1^H-^1^H TOCSY spectra of strain *Shewanella eurypsychrophilus* YLB-09 obtained using a Bruker Avance III 600 MHz NMR spectrometer at 25℃ and pH 7.4**.

**Figure S3.**

**
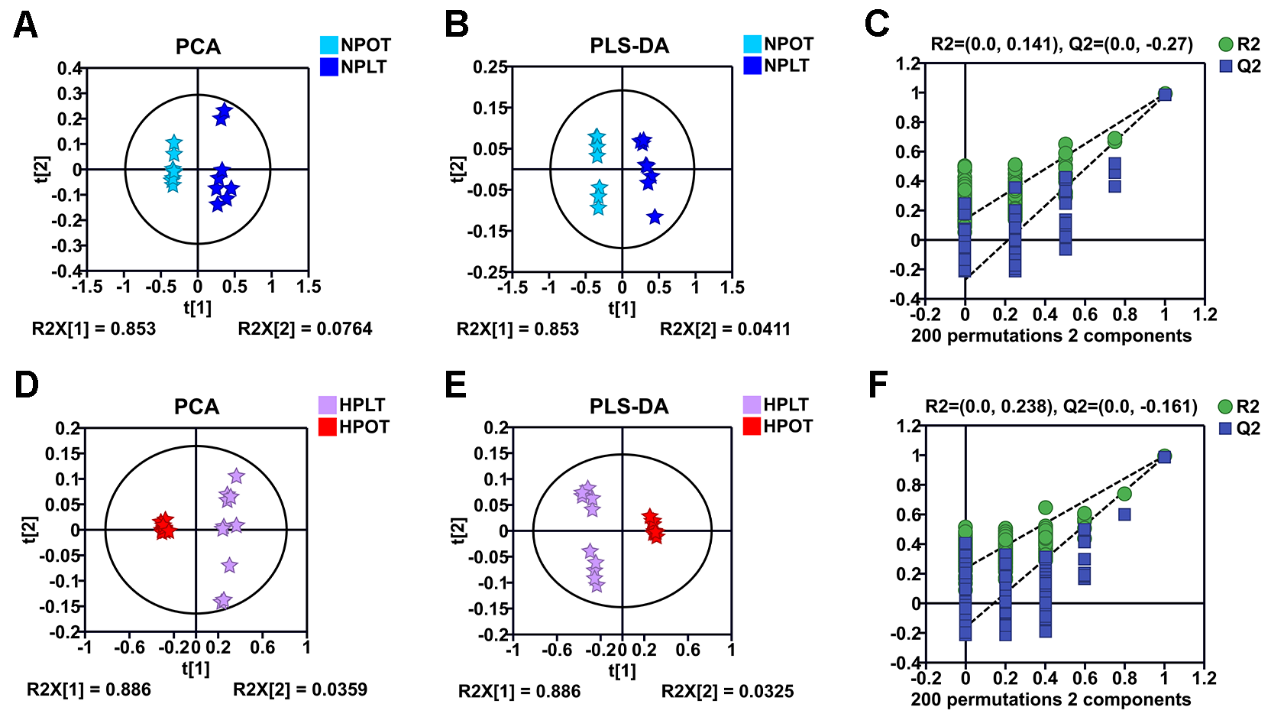
**

**Figure S3.** **Multivariate statistical analyses for NMR data of YLB-09 cells under low temperature conditions.** (A-C) Scores plots of PCA and PLS-DA models of NPLT *vs.* NPOT, and cross-validation plot of the PLS-DA model. (D-F) Scores plots of PCA and PLS-DA models of HPLT *vs.* HPOT, and cross-validation plot of the PLS-DA model.

**Figure S4.**

**
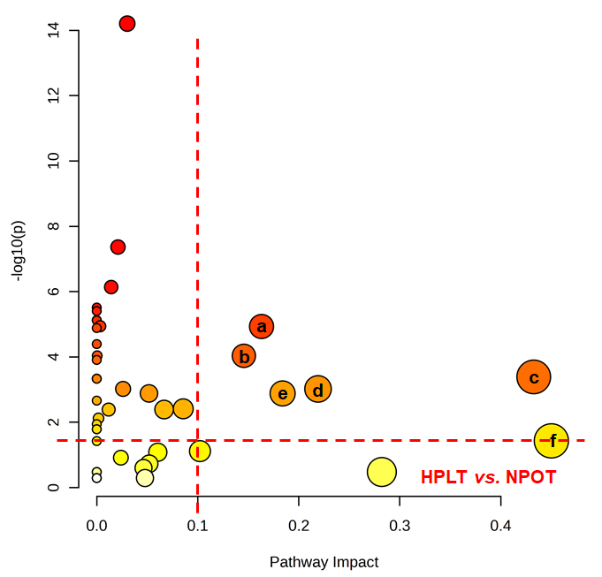
**

**Figure S4.** **Metabolic pathway analysis conducted on YLB-09 cells exposed to high-pressure and low temperature conditions (HPLT *vs.* NPOT).** Significantly altered pathways were identified using the thresholds of pathway impact value (PIV) > 0.1 and *p* < 0.05 via the pathway analysis module of MetaboAnalyst 5.0 web server. The significantly altered pathways were as follows: a-Citrate cycle (TCA cycle); b-Pyrimidine metabolism; c-Glycine, serine and threonine metabolism; d-Glycerolipid metabolism; e-Methane metabolism; f-Alanine, aspartate and glutamate metabolism.

**Figure S5.**

**
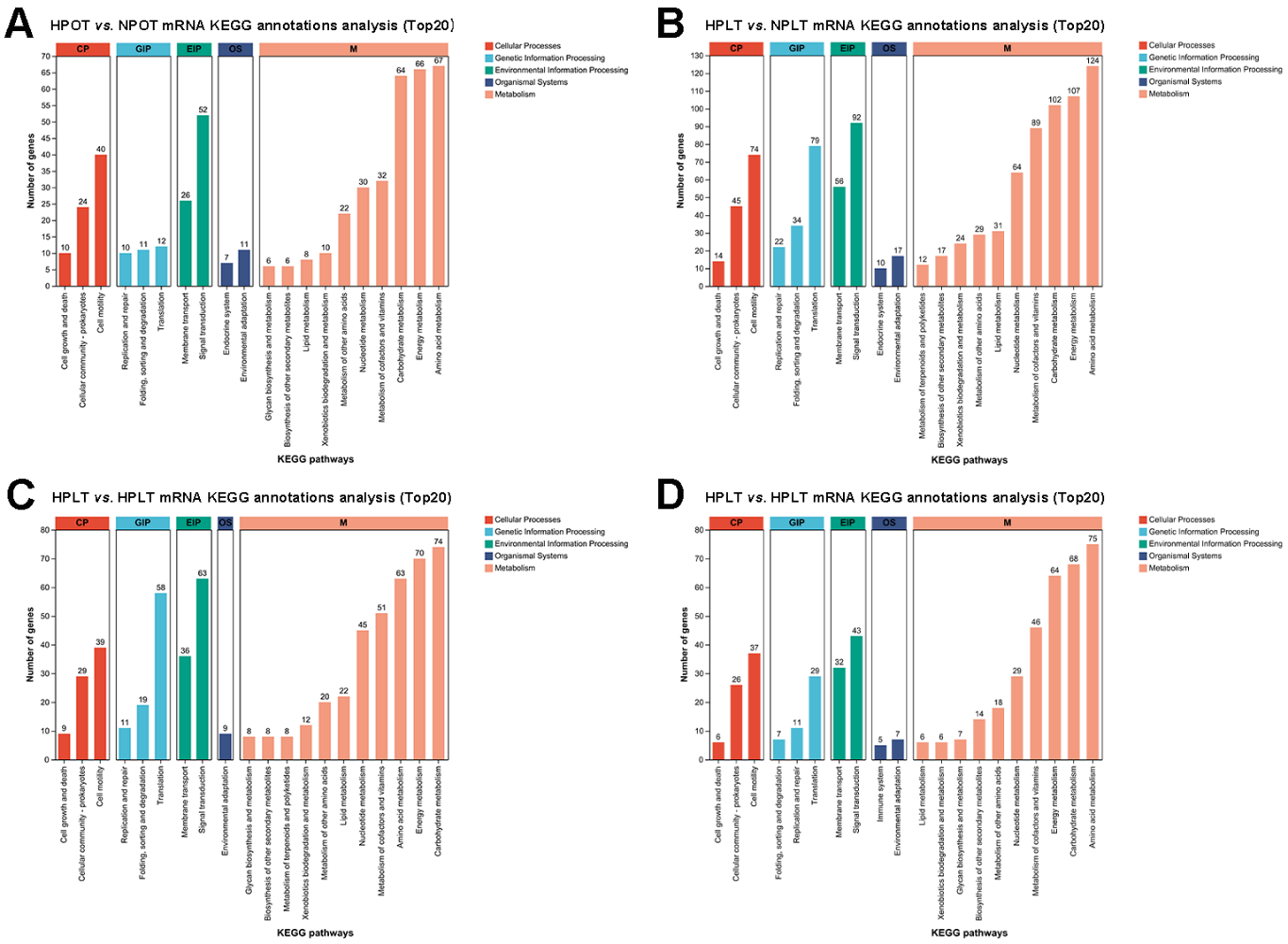
**

**Figure S5. Graphical results of the results of KEGG enrichment analysis of differentially expressed genes under high pressure and low temperature conditions.** (A) HPOT *vs.* NPOT, (B) HPLT *vs.* NPLT, (C) NPLT *vs.* NPOT, (D) HPLT *vs.* HPOT.

**Figure S6.**

**A**

**
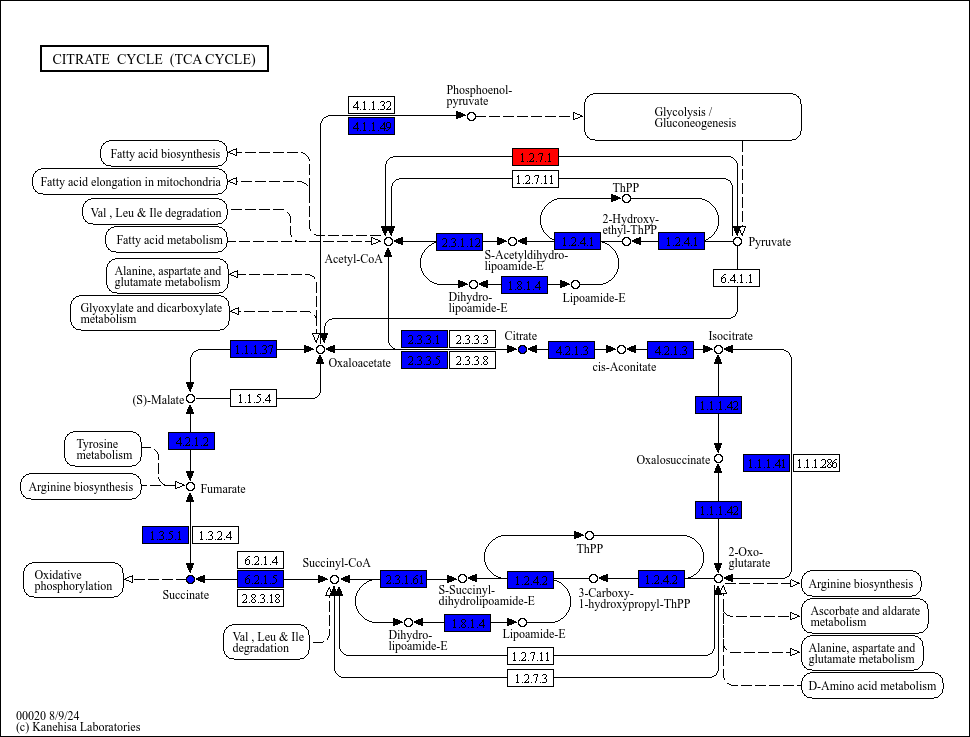
**

**B**


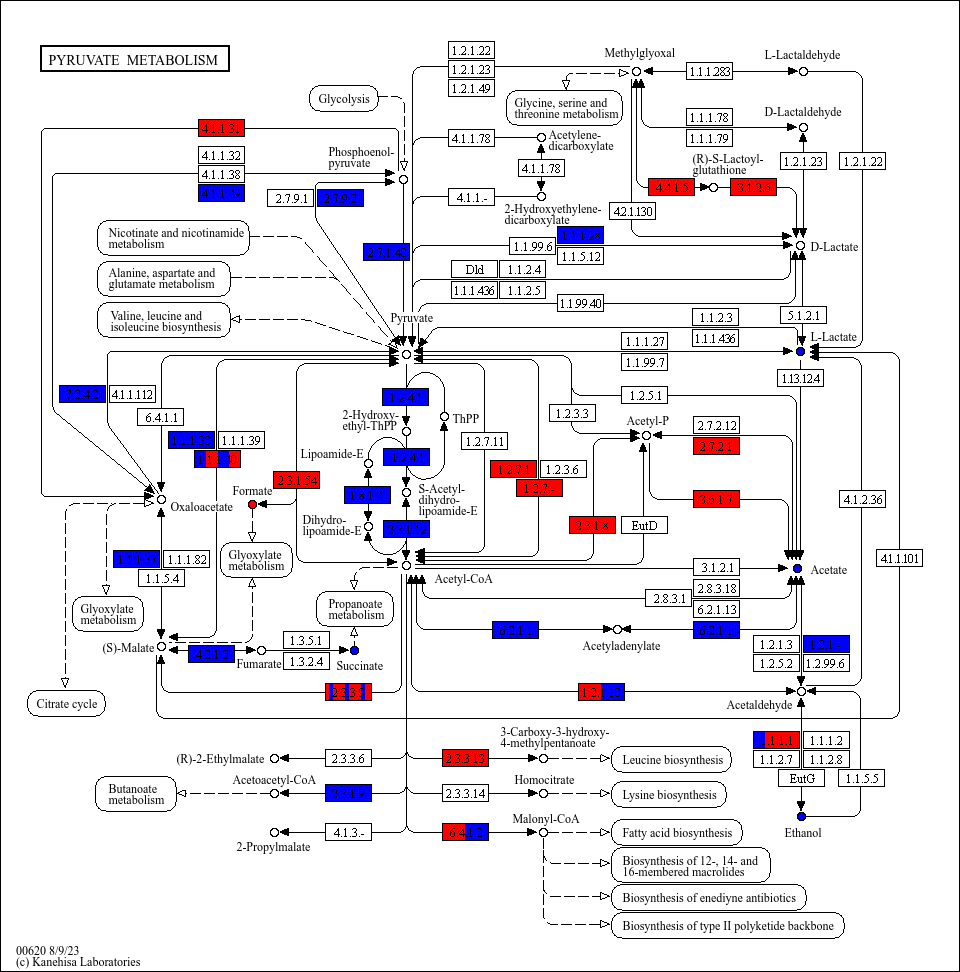


**C**


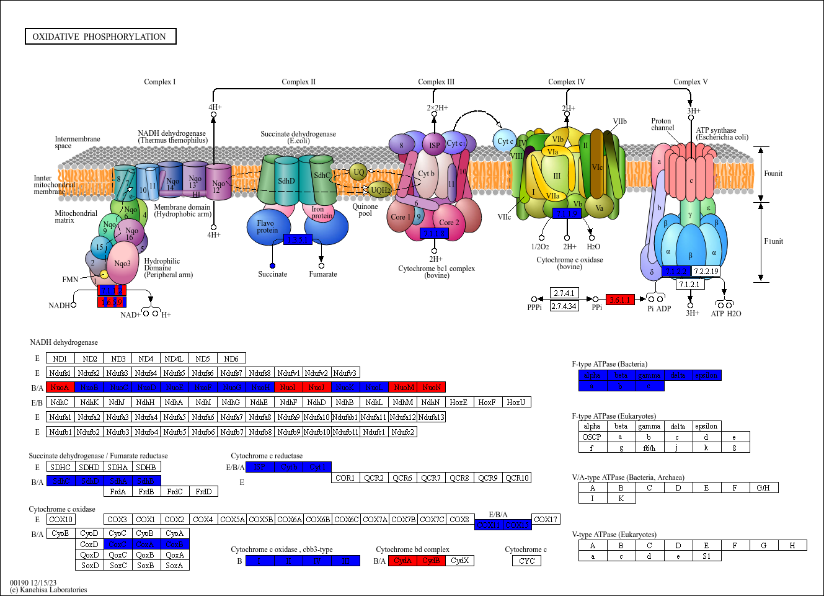


**D**


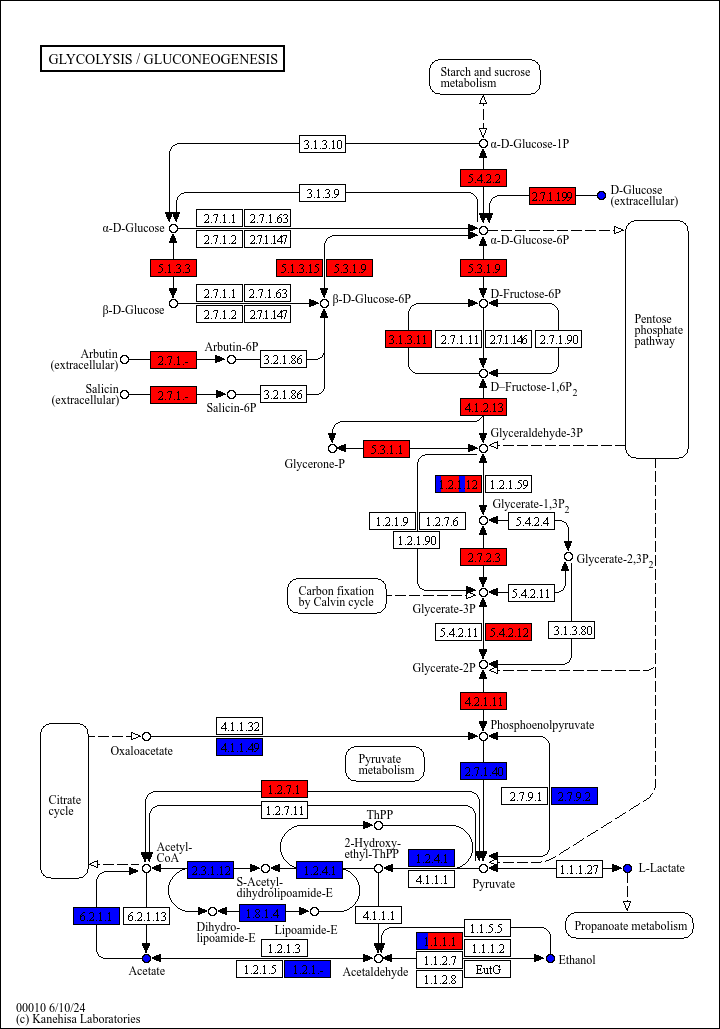


**Figure S6. DEGs annotation of strain YLB-09 undergoes regulated metabolic pathways under high pressure.** This figure was created based on the DEGs obtained from YLB-09 cells. (A) Citrate cycle (TCA cycle), (B) Pyruvate metabolism, (C) Oxidative phosphorylation, (D) Glycolysis / Gluconeogenesis. Red, up-regulated; blue, down-regulated.

**Figure S7.**

**
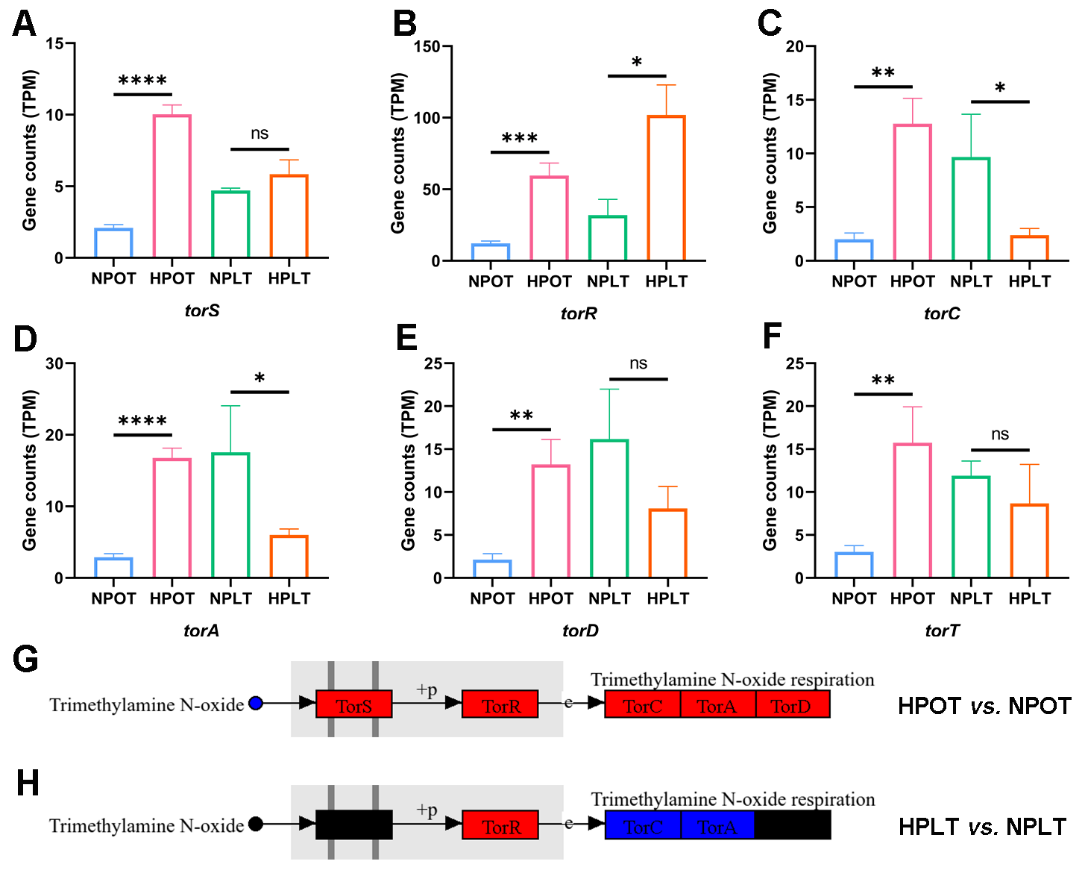
**

**Figure S7. Expression of genes associated with TMAO respiration at high pressure and low temperature.** (A) *torS*, (B) *torR*, (C) *torC*, (D) *torA*, (E) *torD*, (F) *torT*, (G) HPOT *vs.* NPOT, (H) HPLT *vs.* NPLT. Red, up-regulated; blue, down-regulated; black, no significance.

**Figure S8.**

**A**


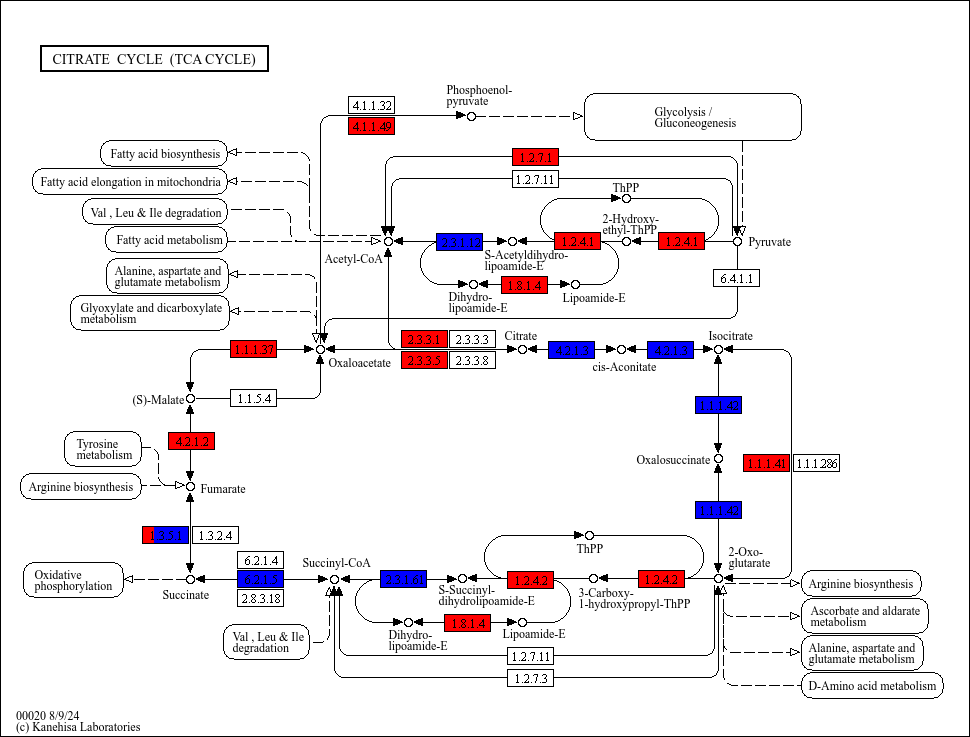


**B**

**
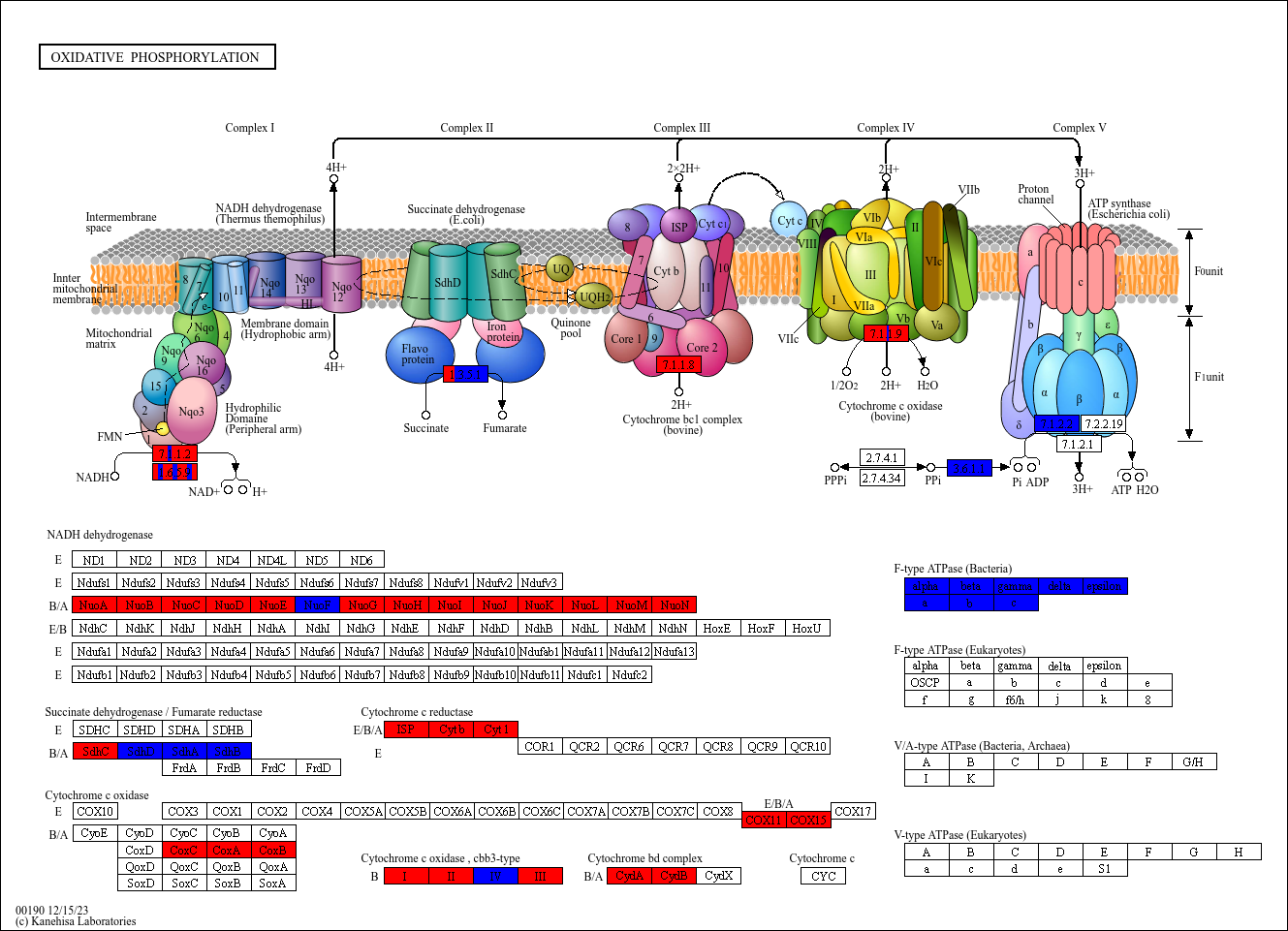
**

**C**

**
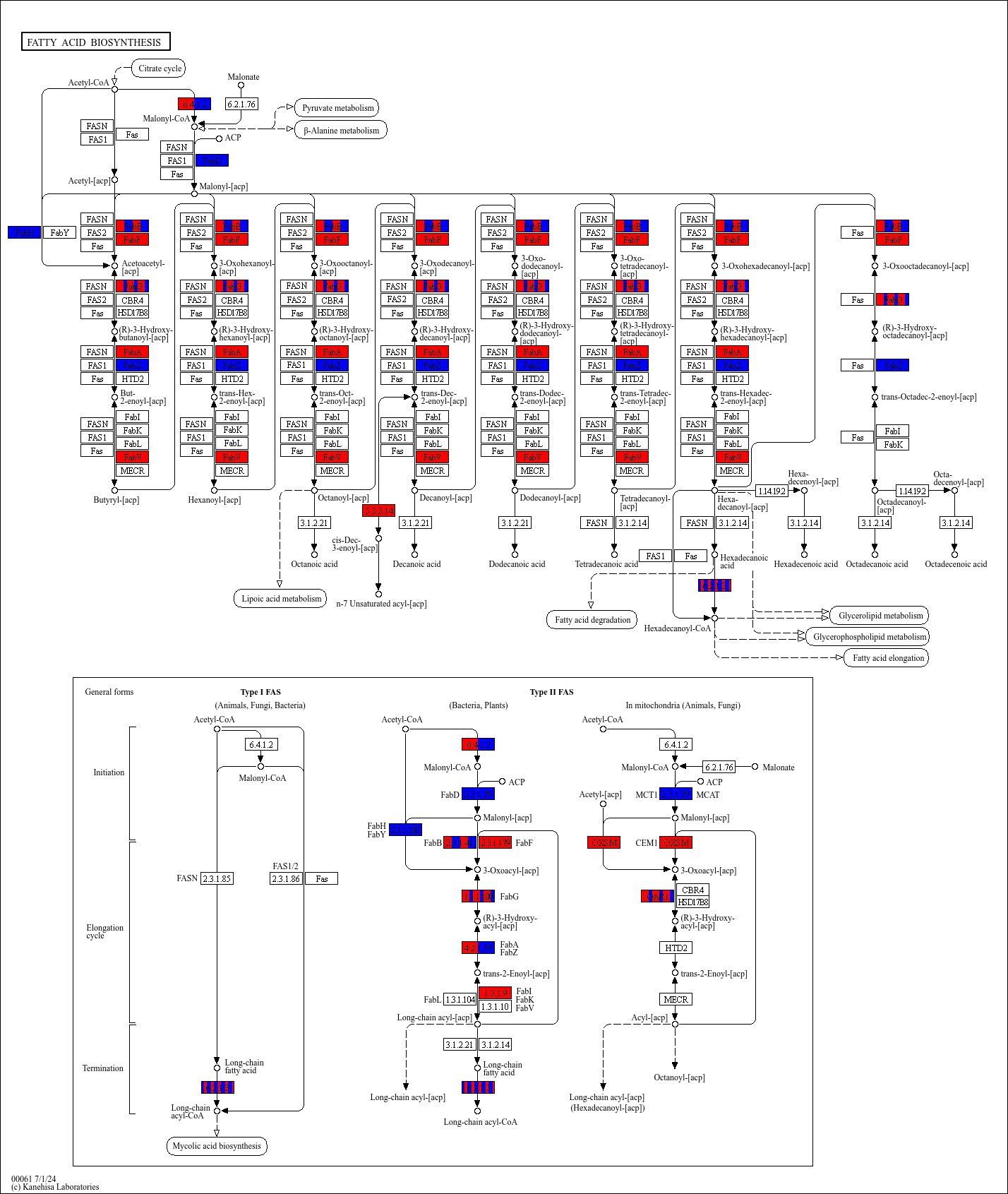
**

**Figure S8. DEGs annotation of strain YLB-09 undergoes regulated metabolic pathways under low temperature.** This figure was created based on the DEGs obtained from YLB-09 cells. (A) Citrate cycle (TCA cycle), (B) Pyruvate metabolism, (C) Fatty acid biosynthesis. Red, up-regulated; blue, down-regulated.

**Table S1. Aqueous metabolites identified from ^1^H-NMR spectra of** ***Shewanella eurypsychrophilus* YLB-09.**

| **Metabolite** | **δ^1^H (ppm) and multiplicity** |
| --- | --- |
| Valine | 0.99 (d), 1.04 (d), 2.26 (m) |
| Lactate | 1.33 (d) |
| Isoleucine | 1.01 (d), 0.94 (t) |
| Alanine | 1.48 (d) |
| Threonine | 1.32 (d), 3.58 (d), 4.24 (m) |
| Citrate | 2.53 (d), 2.67 (d) |
| Glutamate | 2.33 (m), 2.36 (m), 2.04 (m) |
| Acetate | 1.91 (s) |
| Glucose | 3.24 (t), 4.64 (d), 5.23 (d) |
| Aspartate | 2.81 (dd) |
| Glycerol | 3.56 (m), 3.65 (dd), 3.79 (m) |
| Glycine | 3.55 (s) |
| Formate | 8.44 (s) |
| Uracil | 5.79 (d), 7.53 (d) |
| Tyrosine | 6.89 (d), 7.18 (d) |
| Phenylalanine | 7.32 (d), 7.36 (d), 7.43 (t) |
| Oxypurinol | 8.20 (s) |
| Ethanolamine | 3.15 (t), 3.82 (t) |
| 1,7-Dimethylxanthine | 7.82 (s) |
| Leucine | 0.95 (d), 0.97 (d), 1.70 (m) |
| Propylene glycol | 1.14 (d) |
| Ethanol | 1.18 (t) |
| Succinate | 2.38 (s) |
| Creatine | 3.02 (s) |
| Betaine | 3.26 (s) |
| Methanol | 3.35 (s) |
| Serine | 3.95 (dd), 3.98 (dd) |
| Histidine | 7.07 (s), 7.87 (s) |
| Xanthine | 7.86 (d) |
| Imidazole | 7.32 (s), 8.23 (s) |
| Thymidine | 7.64 (s), 6.29 (t), 1.89 (d) |
| Pyridoxine | 2.45 (s), 7.69 (s) |
| Trimethylamine N-oxide | 3.27 (s) |
| Threitol | 3.62 (m), 3.63 (m), 3.69 (m),3.72 (m) |

Multiplicity: s, singlet; d, double; t, triplet; q, quartet; m, multiple; dd, double of double.

**Table S2. Quantitative comparisons of metabolite concentrations between the four groups of YLB-09 cells under low temperature (with or without high pressure).**

| **Metabolites** | **Mean ± SD** | | | | **Significance** | |  |
| --- | --- | --- | --- | --- | --- | --- | --- |
|  | **NPOT** | **NPLT** | **HPOT** | **HPLT** | **NPLT *vs.* NPOT** | **HPLT *vs.* HPOT** | |
| **Amino acid** |  |  |  |  |  |  | |
| Alanine | 21.795±0.679 | 25.192±1.503 | 11.315±0.113 | 18.964±0.811 | ******** | ******** | |
| Aspartate | 5.213±0.270 | 9.622±0.341 | 3.034±0.251 | 6.563±0.833 | ******** | ******** | |
| Glutamate | 28.045±0.870 | 46.649±6.653 | 16.246±0.411 | 28.544±1.932 | ******** | ******** | |
| Glycine | 12.191±0.237 | 14.541±0.686 | 7.636±0.334 | 13.508±1.194 | ******** | ******** | |
| Histidine | 1.894±0.474 | 0.482±0.108 | 0.446±0.182 | 0.765±0.201 | ******** | ****** | |
| Isoleucine | 11.620±0.316 | 12.579±0.666 | 6.364±0.258 | 10.716±0.500 | ****** | ******** | |
| Leucine | 16.372±0.394 | 17.122±0.790 | 8.414±0.168 | 14.754±0.758 | ***** | ******** | |
| Phenylalanine | 9.096±0.792 | 6.572±0.688 | 5.943±0.291 | 6.551±1.058 | ******** | ns | |
| Serine | 10.368±2.180 | 15.306±1.672 | 4.535±0.481 | 13.603±2.238 | ******* | ******** | |
| Threitol | 49.090±1.058 | 97.887±6.132 | 34.057±0.704 | 60.805±3.715 | ******** | ******** | |
| Threonine | 39.128±1.180 | 66.335±5.013 | 22.298±0.541 | 43.123±2.032 | ******** | ******** | |
| Tyrosine | 2.356±0.779 | 2.796±0.289 | 1.137±0.189 | 1.433±0.681 | ns | ns | |
| Valine | 13.703±0.285 | 15.178±0.838 | 8.103±0.321 | 12.811±0.506 | ****** | ******** | |
| **Carbohydrate** |  |  |  |  |  |  | |
| Acetate | 10.188±0.306 | 9.135±0.211 | 9.780±0.119 | 11.176±1.196 | ******** | ****** | |
| Betaine | 1.005±0.126 | 3.378±0.133 | 0.618±0.037 | 1.741±0.162 | ******** | ******** | |
| Citrate | 38.406±0.888 | 38.043±1.811 | 22.661±0.266 | 32.682±2.527 | ns | ******** | |
| Formate | 3.539±0.396 | 6.574±0.851 | 3.926±0.290 | 4.980±0.675 | ******** | ******* | |
| Glucose | 0.053±0.124 | 9.769±1.089 | 0.040±0.060 | 0.012±0.037 | ******** | ns | |
| Lactate | 36.409±2.740 | 36.131±4.770 | 26.127±1.787 | 41.923±4.524 | ns | ******** | |
| Succinate | 1.371±0.108 | 1.629±0.061 | 0.982±0.052 | 2.254±0.343 | ******** | ******** | |
| **Nucleotide** |  |  |  |  |  |  | |
| 1,7-Dimethylxanthine | 0.938±0.462 | 0.679±0.151 | 0.163±0.126 | 0.190±0.154 | ns | ns | |
| Oxypurinol | 2.415±0.710 | 1.865±0.415 | 0.363±0.295 | 0.590±0.342 | ns | ns | |
| Thymidine | 8.327±0.266 | 11.078±0.534 | 4.855±0.106 | 8.207±0.493 | ******** | ******** | |
| Uracil | 6.350±1.560 | 6.483±0.482 | 1.219±0.235 | 3.266±0.938 | ns | ******** | |
| Xanthine | 3.622±0.823 | 1.048±0.432 | 0.586±0.272 | 1.135±0.519 | ******** | ****** | |
| **Others** |  |  |  |  |  |  | |
| Creatine | 2.096±0.178 | 3.471±0.183 | 0.968±0.072 | 2.161±0.425 | ******** | ******** | |
| Ethanol | 39.856±3.523 | 35.142±1.652 | 29.289±1.858 | 37.870±2.119 | ****** | ******** | |
| Ethanolamine | 43.413±0.606 | 74.594±3.424 | 14.183±0.492 | 26.797±1.603 | ******** | ******** | |
| Glycerol | 64.294±2.121 | 83.131±4.057 | 59.948±0.839 | 71.841±4.908 | ******** | ******** | |
| Imidazole | 1.370±0.442 | 1.577±0.544 | 0.231±0.124 | 0.671±0.260 | ns | ******* | |
| Methanol | 3.802±0.898 | 46.521±5.264 | 2.781±0.733 | 4.118±0.558 | ******** | ******* | |
| Propylene glycol | 12.967±0.712 | 12.280±0.931 | 9.819±0.452 | 12.594±0.649 | ns | ******** | |
| Pyridoxine | 1.351±0.582 | 0.546±0.234 | 0.282±0.219 | 0.818±1.146 | ****** | ns | |
| TMAO | 5.215±0.216 | 5.794±2.889 | 2.844±0.052 | 4.893±1.638 | ns | ****** | |

Note: Statistical significances of differences in metabolite concentration were analyzed by independent sample *t*-test: ns, *p* > 0.05; *, *p* < 0.05; **, *p* < 0.01; ***, *p* < 0.001; ****, *p* < 0.0001. Red, increase in metabolite concentration; blue, decreases in metabolite concentration.
